# Electrodeposition of Pt-Decorated Ni(OH)_2_/CeO_2_ Hybrid as Superior Bifunctional Electrocatalyst for Water Splitting

**DOI:** 10.34133/2020/9068270

**Published:** 2020-12-15

**Authors:** Huanhuan Liu, Zhenhua Yan, Xiang Chen, Jinhan Li, Le Zhang, Fangming Liu, Guilan Fan, Fangyi Cheng

**Affiliations:** Key Laboratory of Advanced Energy Materials Chemistry (Ministry of Education), Renewable Energy Conversion and Storage Center, College of Chemistry, Nankai University, Tianjin 300071, China

## Abstract

The facile synthesis of highly active and stable bifunctional electrocatalysts to catalyze water splitting is attractive but challenging. Herein, we report the electrodeposition of Pt-decorated Ni(OH)_2_/CeO_2_ (PNC) hybrid as an efficient and robust bifunctional electrocatalyst. The graphite-supported PNC catalyst delivers superior hydrogen evolution reaction (HER) and oxygen evolution reaction (OER) activities over the benchmark Pt/C and RuO_2_, respectively. For overall water electrolysis, the PNC hybrid only requires a cell voltage of 1.45 V at 10 mA cm^−2^ and sustains over 85 h at 1000 mA cm^−2^. The remarkable HER/OER performances are attributed to the superhydrophilicity and multiple effects of PNC, in which Ni(OH)_2_ and CeO_2_ accelerate HER on Pt due to promoted water dissociation and strong electronic interaction, while the electron-pulling Ce cations facilitate the generation of high-valence Ni OER-active species. These results suggest the promising application of PNC for H_2_ production from water electrolysis.

## 1. Introduction

Hydrogen is the ideal alternative of fossil fuels as an important clean energy and industrial material [1, 2]. Water electrolysis using electricity from renewable energy is a facile and attractive technique to produce hydrogen with high purity [3, 4]. Electrocatalysts play the paramount role in overcoming the slow dynamics of both hydrogen and oxygen evolution reactions (HER and OER) [5–7]. While Pt and Ru/Ir-based compounds are highly efficient to catalyze HER and OER, respectively [8, 9], their fancy price, extremely low elemental reserves, and unsatisfactory durability limit large-scale applications [10–12], calling for strategies to improve stability and reduce noble metal loading [13–15].

In alkaline media, although Pt is the benchmarking HER catalyst, its catalytic activity is still restrained by water dissociation due to the lack of oxyphilic surfaces to cleave the O−H bond of H_2_O [16, 17]. The synergistic effect mechanism of Pt and Ni(OH)_2_ has been indicated in previous studies: Ni(OH)_2_ promotes the water dissociation and the generation of hydrogen atoms, which are absorbed at Pt sites nearby to recombine into hydrogen molecules [18–24]. In addition, using a bifunctional catalyst could avoid different synthetic processes of electrocatalysts and simplify the water electrolysis device to lower the cost [25]. However, Pt/Ni(OH)_2_ catalysts do not favor the OER due to sluggish kinetics. The electronic structure of Ni could be modulated through the integration of CeO_2_ in Ni(OH)_2_, which could further promote to form *γ*-NiOOH, making the Ni(OH)_2_/CeO_2_ a superb OER catalyst [26, 27]. Meanwhile, Ce can impose large electronic perturbation to Pt nanoparticles, which may enhance the HER ability of the neighboring Pt sites [28]. Therefore, the Pt-Ni(OH)_2_-CeO_2_ hybrid has great potential for electrocatalysis of both HER and OER but has not been reported previously. Besides, the Pt-based hybrid catalysts usually suffer from complicated preparation procedures, particle agglomeration, and weak adhesion between catalyst and substrate [29–31].

In this study, we report a facile electrodeposition method to synthesize ultrafine Pt nanoparticles (NPs) decorated Ni(OH)_2_/CeO_2_ nanosheets (abbreviated as PNC) via anion intercalation and cathodic electrodeposition method. The Pt NPs with an average size of 3.1 nm are homogeneously deposited on the Ni(OH)_2_/CeO_2_ (abbreviated as NC) nanosheets, which could increase the exposure area of active sites and improve the utilization of Pt. Additionally, the mosaic-structured NC nanosheets not only effectively catalyze the OER but also enhance the HER activity of Pt due to electronic modulation and promoted dissociation of water. Furthermore, anion intercalation and in situ growth of the hybrid catalyst on graphite construct interfaces with strong adhesion, resulting in efficient electron transportation and enhanced stability. As a result, the prepared PNC electrocatalyst exhibits superior activity and stability to catalyze overall water electrolysis.

## 2. Result and Discussion

**Figure 1:**
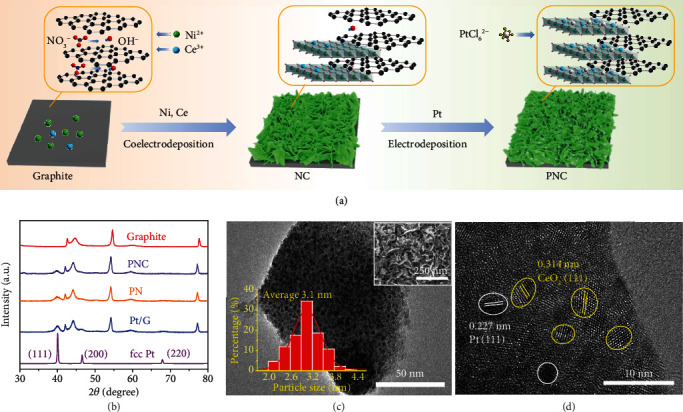
Synthesis and characterization of PNC. (a) Preparation diagram of PNC electrocatalyst on graphite. (b) XRD analysis of Pt/G, PN, and PNC. (c) TEM images of PNC. The inset shows the SEM image of PNC and the size distribution of Pt and CeO_2_ NPs. (d) HRTEM images of PNC.

The PNC catalyst was synthesized through a two-step cathodic electrodeposition process as illustrated schematically in Figure 1(a). Graphite substrate was pretreated by applying a positive potential to allow nitrate ions inserting into graphite layers to enhance the interface of catalyst and graphite. The potential was reversed to realize the cathodic coelectrodeposition of cerium dioxide and nickel hydroxide on graphite. Subsequently, Pt NPs were deposited on the NC nanosheets to obtain the PNC hybrid electrode. In this two-step deposition process, the Pt loading can be reduced and the exposure of Pt is increased to enhance the utilization of Pt, outperforming the reverse sequence when Pt is deposited prior to transition metal hydroxide. The involved reactions in the preparation of PNC are described by equations (1)–(4). For comparison, Pt/G and Pt-Ni(OH)_2_ (abbreviated as PN) electrodes were obtained through a similar process.

(1) $$\begin{array}{c} {{\text{NO}}_{3}}^{-}+{\text{H}}_{2}\text{O}+2{\text{e}}^{-}⟶2\text{O}{\text{H}}^{-}+{{\text{NO}}_{2}}^{-} \end{array}$$

(2) $$\begin{array}{c} {\text{Ni}}^{2+}+2\text{O}{\text{H}}^{-}⟶\text{Ni}{\left(\text{OH}\right)}_{2}↓ \end{array}$$

(3) $$\begin{array}{c} 4\text{C}{\text{e}}^{3+}+12\text{O}{\text{H}}^{-}+{\text{O}}_{2}⟶4\text{Ce}{\text{O}}_{2}↓+6{\text{H}}_{2}\text{O} \end{array}$$

(4) $$\begin{array}{c} {{\text{PtCl}}_{6}}^{2-}+2{\text{H}}_{2}\text{O}⟶\text{Pt}↓+4{\text{H}}^{+}+6\text{C}{\text{l}}^{-}+{\text{O}}_{2}↑ \end{array}$$

Figure 1(b) shows the X-ray diffraction (XRD) patterns of the prepared Pt, PN, and PNC. In addition to the reflections of graphite substrate, the diffraction peaks centered at 2*θ* of 40.0 and 46.5° correspond to the (111) and (200) planes of the face-centered cubic (fcc) Pt (space group of Fm-3m), respectively. The Ni(OH)_2_ and CeO_2_ signals are not observed in XRD because of weak crystalline state of Ni(OH)_2_ and low content of CeO_2_ (supplementary Figure S1). The morphology and microstructure were investigated by a scanning electron microscope (SEM) and transmission electron microscope (TEM). The prepared PN and PNC are composed of aggregated nanoparticles with interparticle pores and flat nanosheets with a thickness of 5–10 nm, while the control Pt/G is granular (Figure 1(c) inset and supplementary Figure S2). This rimous texture and interlinked lamellar morphology would facilitate the penetration of electrolyte inside the electrode and increase the active site exposure area. Contact angle tests indicate superhydrophilic property of PNC (supplementary Figure S3), which can be attributed to the rimous texture and the presence of O−H bond in hydroxide nanosheets. TEM images (Figure 1(c) and supplementary Figure S4) show uniform dispersion of Pt NPs on the hydroxide support. The average size of Pt NPs is 3.1 nm in PNC, smaller than PN (3.4 nm) and Pt/G (13.2 nm). The superhydrophilic surfaces of NC favor the nucleation and growth of Pt, leading to smaller NPs and uniform distribution [32, 33]. The high dispersion of Pt NPs on the mosaic structure of CeO_2_ and the weak crystallinity of Ni(OH)_2_ would enrich the interface between metal and oxide/hydroxide. High-resolution TEM (HRTEM) reveals lattice spacings of 0.314 and 0.227 nm (Figure 1(d)), which are separately assigned to the (111) planes of fcc CeO_2_ and fcc Pt. Energy dispersive spectroscopy (EDS) mapping further confirms the homogenous distribution of Pt, Ni, Ce, and O in PNC (supplementary Figure S5). As detected by EDS and inductively coupled plasma atomic emission spectroscopy (ICP-AES) (supplementary Figure S6), the Pt loadings are 8.8 and 8.2 wt.% in PN and PNC, respectively. The Pt content could be tuned by adjusting the electrodeposition time in H_2_PtCl_6_ solution (supplementary Table S1).

**Figure 2:**
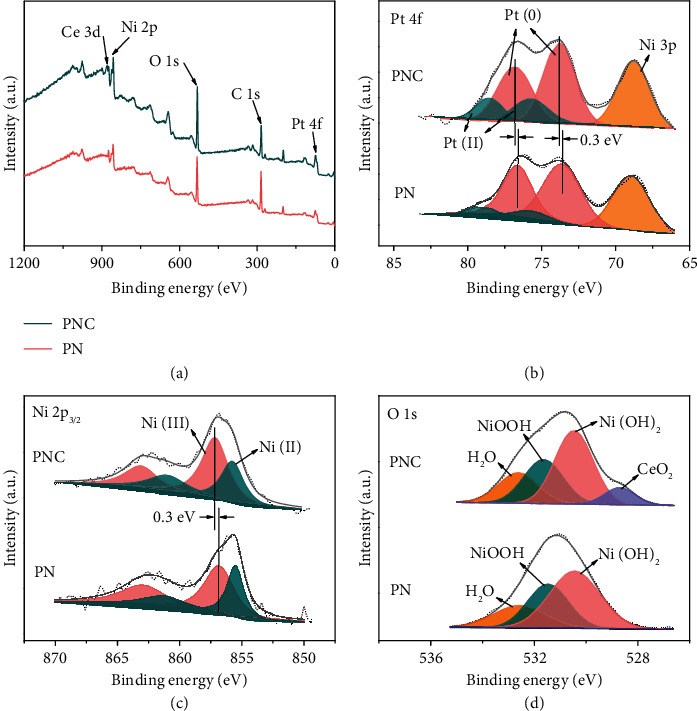
XPS spectra. (a) XPS survey spectra of PN and PNC. (b) Pt 4f spectra. (c) Ni 2p_3/2_ region. (d) O 1s region.

The oxidation states of Pt, Ni(OH)_2_, and CeO_2_were detected by X-ray photoelectron spectroscopy (XPS) spectra (Figure 2). The survey spectra confirm the presence of Pt, Ni, and O in PN and additional Ce in PNC (Figure 2(a)). As shown in Figure 2(b), Pt 4f spectra display two pairs of peaks (4f_7/2_ and 4f_5/2_), corresponding to dominant Pt(0) (75.9%) and surface Pt(II) (24.1%) in PNC. For PN, the atomic ratio of Pt(0) and Pt(II) is 90.7% and 9.3%, respectively. The higher Pt(II) content and 0.3 eV positive shift of Pt(0) peaks in PNC compared to PN suggest partial oxidation of Pt and electronic interaction between Pt and NC [28, 34, 35]. The peak shift also indicates a downshift of the d-band center of Pt in PNC, which could favor hydrogen adsorption on Pt and accelerate the recombination of H_ad_ to form H_2_ [36–38].

As discerned by Ni 2p XPS spectra (Figure 2(c)), both Ni (II) (855.6 eV) and Ni (III) (857.1 and 856.8 eV) exist in PNC and PN. There is a 0.3 eV positive shift of Ni(III) in PNC, indicating electron transfer from Ni to Ce. The electron-pulling ability of ceria would accelerate the generation of NiOOH active sites for OER. In addition, the coexistence of Ni 3p peak in the Pt 4f region confirms Ni(II) species in Ni(OH)_2_ [39]. The O 1s spectrum (Figure 2(d)) of PNC shows deconvoluted peaks at 528.6, 530.5, 531.6, and 532.6 eV assigned to CeO_2_, Ni(OH)_2_, NiOOH, and H_2_O, respectively [26, 40]. Collecting XPS results suggest strong electron interaction and synergy effect between Pt and Ce-Ni oxide/hydroxide in PNC, which would favor the formation of more electrocatalytically active species [26, 41].

**Figure 3:**
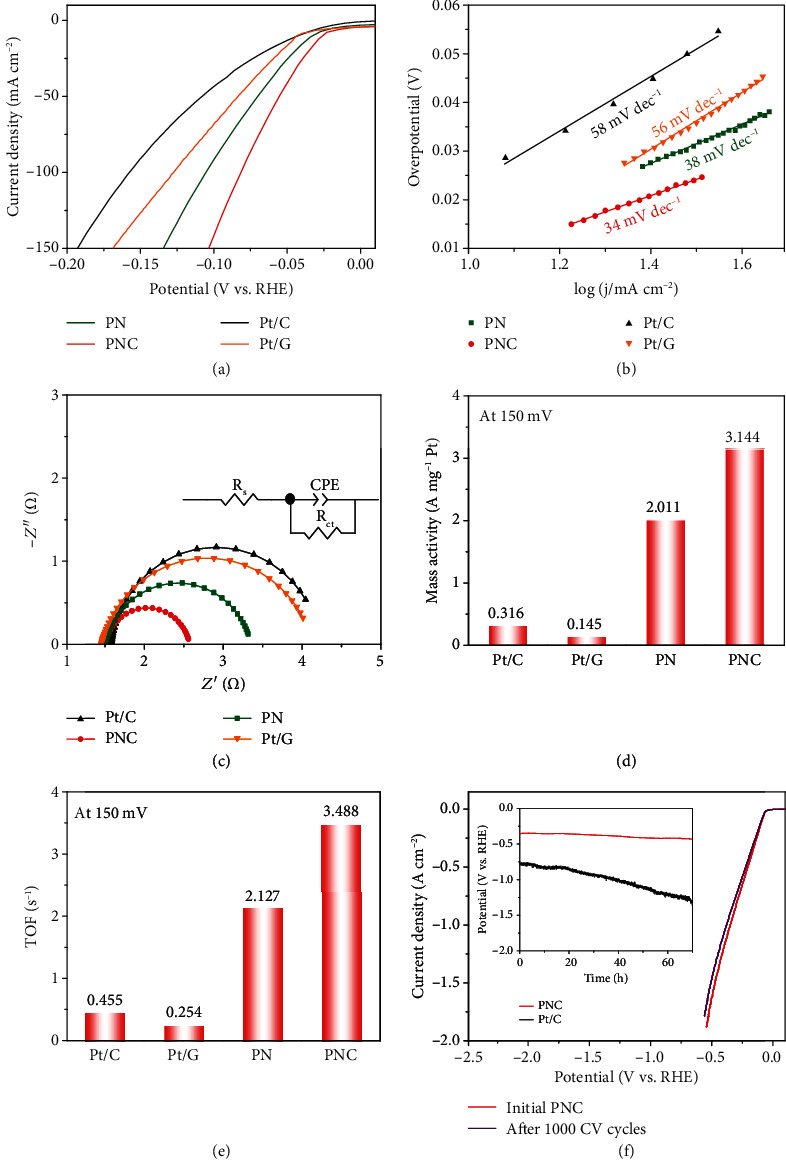
HER performance. (a) HER LSV curves and (b) Tafel plots of Pt/C, Pt/G, PN, and PNC in 1.0 M KOH. (c) EIS Nyquist plots. (d) Pt mass activities at the overpotential of 150 mV. (e) Turnover frequency (TOF) deduced from the LSV curves at overpotential of 150 mV. (f) LSV curves of PNC before and after 1000 cycles. The inset shows the chronopotentiometry (*E*‐*t*) curves of PNC and Pt/C at 1000 mA cm^−2^.

The electrocatalytic HER performances were investigated by linear sweep voltammetry (LSV). As shown in Figure 3(a), the onset potential of PNC is near 0 mV and it needs only 76 mV overpotential at 100 mA cm^−2^, significantly outperforming PN (105 mV), Pt/G (124 mV), and 20 wt.% Pt/C (153 mV). At a high applied current density, rapid generation and release of bubbles occur while no catalyst desquamating from the electrode surface is observed for PNC (supplementary Movie S1). The results indicate superior mechanical stability of the electrodeposited hybrid material, which is favorable for industrial application operating under large current density.

Tafel slope is a figure-of-merit to assess HER kinetics of electrocatalysts. In Figure 3(b), the PNC represents a Tafel slope of 38 mV dec^−1^, which is much smaller than PN, Pt/C, and Pt/G (45, 56, and 58 mV dec^−1^) and indicates rapid HER kinetics in PNC (Tafel-Volmer mechanism). The HER process in alkaline condition includes the Volmer adsorption step (H_2_O + e^−^⟶H_ad_ + OH^−^) combined with the Heyrovsky desorption step (H_ad_ + H_2_O + e^−^⟶H_2_ + OH^−^) or the Tafel recombination step (H_ad_ + H_ad_⟶H_2_) [42, 43]. In alkaline media, the Volmer step is suggested to determine the HER rate of Pt, due to the sluggish dissociation of water [44, 45]. For the PNC hybrid, the metal (hydro)oxides are efficient to split O−H bond and accelerate the Volmer step, leading to an improvement in HER kinetics. The electrode kinetics of the HER process is further investigated by conducting the EIS experiments, where the equivalent circuit was composed of an electrolyte resistance (*R*_s_) in series with a parallel connection of a constant phase element (CPE) and a charge-transfer resistance (*R*_ct_) [46]. From the Nyquist plots (Figure 3(c)) recorded at the potential of −0.026 V vs. RHE, PNC has a smaller *R*_ct_ compared to Pt/C, Pt/G, and PN. The enhanced charge transfer is attributed to the interfacial electron interaction, plate-like nanostructure with shorter diffusion pathway, and rimous texture that allows facile electrolyte access.

The mass activities were calculated to evaluate the utilization ratio of Pt in the catalysts (Figure 3(d)). At the overpotential of 150 mV, the PNC electrode achieves a remarkable current density of 3.144 A mg^−1^_Pt_, which is almost 10 and 1.6 times those of the counterpart 20 wt.% Pt/C and PN (0.316 and 2.011 A mg^−1^_Pt_). The improvement of the mass activity of Pt could be ascribed to the high dispersion and ultrafine particle of Pt. The highest HER activities of PN and PNC are attained when the Pt mass loadings are 8.8 wt.% and 8.2 wt.%, respectively (supplementary Figure S7). Moreover, cyclic voltammetry (CV) curves at different scan rates are measured to obtain the electrochemical double-layer capacitance (*C*_dl_) for evaluating the electrochemical active surface areas (ECSAs) (supplementary Figure S8). The current densities are normalized by ECSA or mass of the catalyst to determine specific activity or mass activity at an overpotential of 150 mV. Clearly, PNC delivers overwhelming values as compared to PN, Pt/C, and Pt/G (supplementary Figure S9). The intrinsic catalytic activity can be further evaluated by the turnover frequency (TOF) [47]. Assuming Pt as HER active site, the calculated TOF of PNC is 3.488 s^−1^ at overpotential of 150 mV, significantly outperforming PN (2.127 s^−1^), Pt/G (0.254 s^−1^), and Pt/C (0.455 s^−1^) (Figure 3(e)). Besides, PNC exhibits superior HER performance as compared with those of previously reported electrocatalysts (supplementary Table S2).

The long-term catalytic durability was investigated by CV sweeping for 1000 cycles, indicating negligible activity loss of the PNC electrode. The overpotential only slightly increases by 21 mV at 1000 mA cm^−2^, surpassing that of Pt/C (116 mV) (Figure 3(f) and supplementary Figure S10). The catalytic stability of PNC was further tested by chronopotentiometry at a large current density of 1000 mA cm^−2^. As shown in Figure 3(f) inset, PNC shows only a very small increase of overpotential after 70 h test, while Pt/C experiences an apparent increase in polarization. Microscopy imaging on cycled electrode reveals that PNC retains its texture and morphology without noticeable particle agglomeration or inhomogeneous elemental distribution, irrespective of a particle size increase of Pt NPs from 3.1 to 5.6 nm (Supplementary Figure S11). Furthermore, the XRD analysis suggests no phase change, while the XPS results show essentially preservation of the oxidation states of Pt and Ce states but an increase in the proportion of Ni (II) species (supplementary Figure S12). The confinement of Pt NPs in hydroxide and the firm adhesion of electrodeposit on graphite could account for the remarkable catalytic and structural stability at large current density.

**Figure 4:**
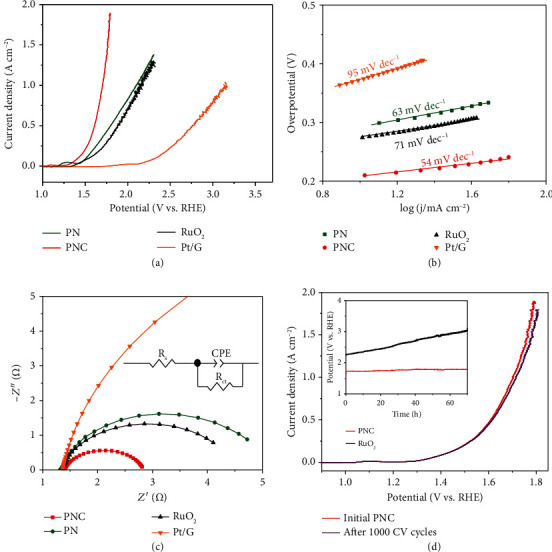
OER performance. (a) OER LSV curves and (b) Tafel plots of Pt/G, PN, PNC, and RuO_2_ in 1.0 M KOH. (c) EIS Nyquist plots. (d) LSV curves of PNC before and after 1000 OER cycles. The inset shows the chronopotentiometry curves of PNC and RuO_2_ at 1000 mA cm^−2^.

The OER performances were also assessed by LSV curves (Figure 4(a)). The PNC exhibits a lower overpotential of 186 mV (100 mA cm^−2^), as compared to PN (279 mV), RuO_2_ (321 mV), and Pt/G (1094 mV). The current fluctuation in the LSV curves of RuO_2_ results from the formation of large O_2_ bubbles. Differently, PNC manifests enhanced and stable OER performance without rigorous fluctuation or large bubble generation at high current density (supplementary Movie S2). Furthermore, the Tafel slope of PNC is 54 mV dec^−1^, lower than that of PN (63 mV dec^−1^), RuO_2_ (71 mV dec^−1^), and Pt/G (95 mV dec^−1^) (Figure 4(b)). Remarkably, the OER activity of PNC is among the highest referencing results (supplementary Table S3).

Figure 4(c) shows the EIS data to assess the OER kinetics. At the potential of 1.474 V vs. RHE, a much lower *R*_ct_ value (1.4 *Ω*) was obtained on PNC, in comparison to PN (3.7 *Ω*), Pt/G (18.4 *Ω*), and RuO_2_ (3.1 *Ω*). Continuous CV sweeping was tested to investigate the OER durability of PNC (Figure 4(d)). The OER overpotential at 1000 mA c﻿m^−2^ increases only 12 mV after 1000 cycles, revealing insignificant change as compared with RuO_2_ (343 mV) (supplementary Figure S13). In addition, the chronopotentiometric response (Figure 4(d) inset) shows unconspicuous decay at 1000 mA cm^−2^ for 70 h, further indicating superior OER catalytic stability of PNC. A combination of SEM, TEM, EDS, and XRD characterizations indicates no apparent change in morphology, composition, and phase but a slight increase of particle size after extended OER electrocatalysis on PNC (supplementary Figures S14 and 15). Of note is that an increased ratio of NiOOH, which has been regarded as the active center of OER [26, 48], can be analyzed by XPS results (supplementary Figure S15d).

**Figure 5:**
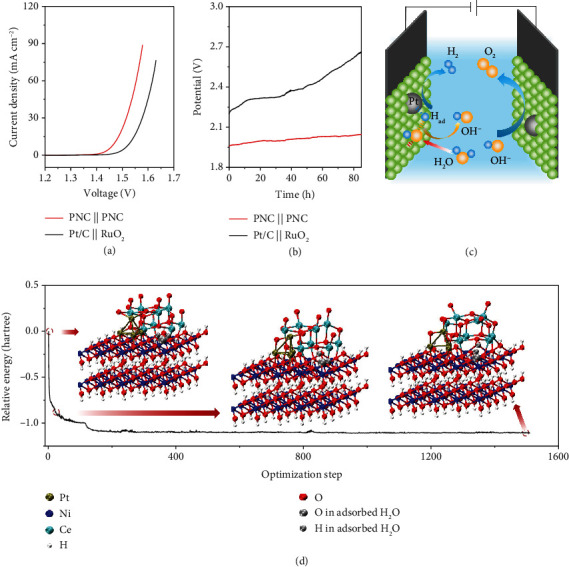
Water splitting performance. (a) LSV curves of the PNC || PNC and Pt/C || RuO_2_ electrolyzers in 1.0 M KOH. (b) Chronopotentiometry curves of PNC || PNC and Pt/C || RuO_2_ electrolyzers at 1000 mA cm^−2^. (c) Illustration of the PNC as a bifunctional electrode for overall water electrolysis. (d) DFT calculated the relative energy diagram of the water dissociation process for PNC. The inset shows the structural diagram of PNC at different water decomposition states.

Based on the excellent performances of PNC for both the HER and the OER, a symmetric two-electrode cell was constructed using PNC as a bifunctional electrode for water electrolysis. As shown in Figure 5(a), the cell delivers a current density of 10 mA cm^−2^ at an applied voltage of 1.5 V, 100 mV lower than that of the benchmark Pt/C || RuO_2_ couple (1.55 V). The PNC hybrid competes favorably with most electrocatalysts reported previously for overall water splitting (supplementary Table S4). Remarkably, in the extended stability test at a large current density of 1000 mA cm^−2^, a constant potential of 2.06 V can be well maintained for 85 h with the faintest degradation (Figure 5(b)). A large amount of bubbles release uniformly from the electrode surfaces without any shedding (supplementary Movie S3). Moreover, the volumes of collected H_2_ and O_2_ match well with the theoretical values as a function of time during electrolysis at 1000 mA cm^−2^ (supplementary Figure S16), indicating nearly 100% faradaic efficiency of PNC || PNC electrolyzer. Excitingly, the PNC-based device can be actuated by a 1.5 V battery for vigorous, stable generation, and release of gas (supplementary Figure S17 and Movie S3), again confirming the high efficiency of PNC to electrolyze water.

Figure 5(c) schematically shows the reaction mechanism of the electrolyzer. We propose that metal hydroxide in PNC promotes the rupture of O−H in water and the generation of adsorbed H atoms (H_ads_), which adsorb on the Pt surface to recombine into hydrogen molecule. Meanwhile, the OH^−^ ions are transformed into O_2_ on the anode with different metal centers [22, 26, 49]. To gain further insight into the water dissociation process, we performed preliminary DFT calculations on the configuration and evolution of PNC hybrid and dissociation energy of H_2_O on the interface (Figure 5(d) and supplementary Movie S4). The water is adsorbed at the interface of Ni(OH)_2_ and CeO_2_, where O is connected with the Ni atom in Ni(OH)_2_ and H forms a weak hydrogen bond interaction with O in CeO_2_. These interactions jointly promote the activation of water, resulting in the fracture of O−H bond in water and the formation of the final structure in Figure 5(d). The process is exothermic (about −2.74 eV), indicating that water dissociation can proceed spontaneously at this interface. Accordingly, the superior performance of PNC can be attributed to the synergistic effect among Pt, Ni(OH)_2_, and CeO_2_ in the hybrid, together with the beneficial texture, microstructure, and wettability.

## 3. Conclusion

In summary, we report the electrodeposition of Pt-Ni(OH)_2_-CeO_2_ hybrid on graphite and its application for efficient electrocatalysis of water splitting. In 1.0 M KOH, the PNC electrode needs only overpotentials of 76 and 186 mV to afford 100 mA cm^−2^ current density for HER and OER, respectively. The turn over frequency and Pt-based mass activity towards HER are significantly improved on PNC than the benchmark Pt/C. Remarkably, when PNC electrode is directly utilized as the anode and cathode, the assembled symmetric water electrolyzer well works at a low applied cell voltage of 1.45 V. Additionally, respectable stability of PNC is attained at 1000 mA cm^−2^. The superior activity is due to high dispersion of Pt NPs and electronic interaction among Pt, Ni(OH)_2_, and CeO_2_ while the durability is ascribed to strong deposit/graphite adherence, electrode hydrophilicity, and facilitated release of gas bubbles. The results indicate that Pt-decorated hydroxide/oxide electrodeposited on graphite is a promising electrocatalyst for overall water electrolysis.

## 4. Materials and Methods

### 4.1. Material Synthesis

To prepare the PNC hybrid, cathodic galvanostatic electrodeposition was applied and performed using a two-electrode system in a 50 ml electrolytic bath at 25°C. The working and counter electrode are two graphite plates (1 × 2 cm) [26]. The graphite plate was first preprocessed by applying an anodic current of 20 mA cm^−2^ for 300 s to realize NO_3_^−^ intercalation in the interlamination. Then, a cathodic current of −20 mA cm^−2^ was applied for 300 s in a nitrate solution containing 0.09 M Ni(NO_3_)_2_ and 0.01 M Ce(NO_3_)_3_. After that, cathodic depositions at −20 mA cm^−2^ were performed for different time (50, 100, 150, and 200 s) in 0.02 M H_2_PtCl_6_ solution. For comparison, PN was obtained from electrodeposition in 0.1 M Ni(NO_3_)_2_ and 0.02 M H_2_PtCl_6_ using a similar process, while Pt/G was prepared in 0.02 M H_2_PtCl_6_ at 20 mA cm^−2^ for 300 s.

### 4.2. Material Characterizations

A JEOL JSM-7500F microscope was used to obtain SEM images. TEM and HRTEM were carried out on a FEI Talos F200X G2 system equipped with EDS. A Rigaku MiniFlex 600 diffractometer was used to measure the XRD patterns. XPS data were collected with a Kratos Axis Ultra DLD spectrometer. Elemental analysis was obtained on ICP-AES (PerkinElmer Optima 83000).

### 4.3. Electrochemical Measurements

All electrochemical tests were tested by a three-electrode system on AMETEK Parstat 4000 electrochemical workstation in 1.0 M KOH. The electrodeposited electrodes, Hg/HgO, and graphite rod were employed as the working, reference, and counter electrode, respectively. To study the HER performance, LSV curves were tested at 10 mV s^−1^ from −0.8 to −1.6 V vs. Hg/HgO. The OER activity was tested from 0 to 2.0 V vs. Hg/HgO. To test EIS, the frequency range is set from 100 kHz to 0.01 Hz. Water electrolysis was detected in a two-electrode system, and LSV curves were measured from 1.0 to 2.0 V at 10 mV s^−1^. Potentials were reported versus reversible hydrogen electrode (RHE) unless noted, in line with the equation E (RHE) = 0.924 V + E (Hg/HgO). All LSV curves have been iR corrected unless otherwise specified.

The *C*_dl_ values were determined from the double layer region (without faradaic processes) in the CV curves recorded at different scan rates. The ECSA of the prepared catalysts was calculated according to

(5) $$\begin{array}{c} \text{ECSA}=\frac{C_{\text{dl}}}{C_{\text{s}}}, \end{array}$$

where *C*_dl_ is the double-layer capacitance and *C*_s_ is the specific capacitance (0.040 mF cm^−2^) [50].

The TOF was determined by the following equation [51, 52]:

(6) $$\begin{array}{c} \text{TOF}=\frac{J\times A}{2\times \text{F}\times m}, \end{array}$$

where *J* is the current density at a given overpotential (150 mV), *F* is the Faraday constant (96,485 C mol^−1^), *A* is the electrode surface area (1 cm^2^), and *m* is the number of moles of active species on the electrode. The Pt content in PN and PNC was quantified by ICP-AES. In TOF calculation, Pt atoms are counted as the active sites in PN and PNC.

### 4.4. DFT Calculation

Density function theory calculation was performed by using the CP2K package [53]. PBE functional [54] with Grimme D3 correction [55] was used to describe the system. Unrestricted Kohn-Sham DFT has been used as the electronic structure method in the framework of the Gaussian and plane wave method [56, 57]. The Goedecker-Teter-Hutter (GTH) pseudopotentials [58, 59] and DZVPMOLOPT-GTH basis sets [56] were utilized to describe the molecules. A plane-wave energy cut-off of 500 Ry has been employed.

## Supplementary Materials

Supplementary Materials Figure S1: powder XRD patterns of Ni(OH)_2_ and NC scratched down from the Ti mesh. Figure S2: SEM images of Pt/G (a, b), PN (c, d), and PNC (e, f) at low (a, c, e) and high (b, d, f) magnification. Figure S3: contact angles measured on graphite substrate (a) before and (b–d) after the electrodeposition of Pt (b), PN (c), and PNC (d). Figure S4: (a, b) TEM images and particle size distribution of PN. (c, d) TEM images and particle size distribution of Pt/G. Figure S5: elemental mapping images of PNC. Figure S6: EDS spectra of (a) Pt, (b) PN, and (c) PNC. Figure S7: HER polarization curves of (a) PN and (b) PNC hybrid with different Pt loadings. Figure S8: CV curves of (a) Pt/C, (b) electrodeposited Pt, (c) PN, and (d) PNC. (e) The corresponding linear fitting of the current density versus scan rates. (f) Comparison of ECSA. Figure S9: (a) specific and mass activities derived from the HER polarization curves at 150 mV. Figure S10: polarization curves of 20 wt.% Pt/C before and after 1000 HER cycles. Figure S11: (a) SEM, (b) TEM, (c) HRTEM, and (d) elemental mapping images of PNC after 70 h of HER stability test at 1000 mA cm^−2^. Insets (a) and (b) show high-magnification SEM image and size distribution of Pt nanoparticles, respectively. Figure S12: (a) XRD pattern of PNC after HER stability test for 70 h at 1000 mA cm^−2^. High-resolution XPS spectra of (b) Pt 4f, (c) Ni 2p3/2, and (d) O 1s of PNC after stability test. Figure S13: polarization curves of RuO_2_ before and after 1000 OER cycles. Figure S14: (a) SEM, (b) TEM, (c) HRTEM, and (d) elemental mapping images of PNC after 70 h of OER stability test at 1000 mA cm^−2^. Insets (a) and (b) show high-magnification SEM image and size distribution of Pt nanoparticles, respectively. Figure S15: (a) XRD patterns of PNC after OER stability test. High-resolution XPS spectra of (b) Pt 4f, (c) Ni 2p3/2, and (d) O 1s regions of PNC after stability test. Figure S16: generated and theoretical volumes of H_2_ and O_2_ over time at a constant current density of 1000 mA cm^−2^ in PNC || PNC electrolyzer. Figure S17: digital photographs of the PNC || PNC electrolyzer powered by a 1.5 V battery. Table S1: Pt contents in PN and PNC with different electrodeposition time in the H_2_PtCl_6_ precursor solutions. Table S2: the comparison of HER performances of PNC with other electrocatalysts. Table S3: the comparison of OER performances of PNC with other electrocatalysts. Table S4: comparison of water splitting performance of PNC electrocatalyst with other bifunctional electrocatalysts reported before. Movie S1: the formation and release of bubbles on the PNC electrode surface for HER at different current densities. Movie S2: the formation and release of bubbles on the PNC electrode surface for OER at different current densities. Movie S3: the process of water splitting at different densities and the gas generation and release on electrode surface when the PNC || PNC electrolyzer is powered by a 1.5 V battery. Movie S4: the movie of DFT calculated the water dissociation process on the PNC interface.
