# Supplementary material for: Electrodeposition of Pt-Decorated Ni(OH)2/CeO2 Hybrid as Superior Bifunctional Electrocatalyst for Water Splitting
Source: Research (Wash D C). 2020 Dec 15;2020:9068270. doi: 10.34133/2020/9068270 (PMC7877398; doi:10.34133/2020/9068270)
Supplement: Supplementary Materials — Figure S1: powder XRD patterns of Ni(OH)2 and NC scratched down from the Ti mesh. Figure S2: SEM images of Pt/G (a, b), PN (c, d), and PNC (e, f) at low (a, c, e) and high (b, d, f) magnification. Figure S3: contact angles measured on graphite substrate (a) before and (b–d) after the electrodeposition of Pt (b), PN (c), and PNC (d). Figure S4: (a, b) TEM images and particle size distribution of PN. (c, d) TEM images and particle size distribution of Pt/G. Figure S5: elemental mapping images of PNC. Figure S6: EDS spectra of (a) Pt, (b) PN, and (c) PNC. Figure S7: HER polarization curves of (a) PN and (b) PNC hybrid with different Pt loadings. Figure S8: CV curves of (a) Pt/C, (b) electrodeposited Pt, (c) PN, and (d) PNC. (e) The corresponding linear fitting of the current density versus scan rates. (f) Comparison of ECSA. Figure S9: (a) specific and mass activities derived from the HER polarization curves at 150 mV. Figure S10: polarization curves of 20 wt.% Pt/C before and after 1000 HER cycles. Figure S11: (a) SEM, (b) TEM, (c) HRTEM, and (d) elemental mapping images of PNC after 70 h of HER stability test at 1000 mA cm−2. Insets (a) and (b) show high-magnification SEM image and size distribution of Pt nanoparticles, respectively. Figure S12: (a) XRD pattern of PNC after HER stability test for 70 h at 1000 mA cm−2. High-resolution XPS spectra of (b) Pt 4f, (c) Ni 2p3/2, and (d) O 1s of PNC after stability test. Figure S13: polarization curves of RuO2 before and after 1000 OER cycles. Figure S14: (a) SEM, (b) TEM, (c) HRTEM, and (d) elemental mapping images of PNC after 70 h of OER stability test at 1000 mA cm−2. Insets (a) and (b) show high-magnification SEM image and size distribution of Pt nanoparticles, respectively. Figure S15: (a) XRD patterns of PNC after OER stability test. High-resolution XPS spectra of (b) Pt 4f, (c) Ni 2p3/2, and (d) O 1s regions of PNC after stability test. Figure S16: generated and theoretical volumes of H2 and O2 over time at a consta [file 9068270.f1.zip › SI20201019.docx]

**Supporting Information**

**Electrodeposition of Pt-decorated Ni(OH)_2_/CeO_2_ hybrid as superior bifunctional electrocatalyst for water splitting**

Huanhuan Liu, Zhenhua Yan*, Xiang Chen, Jinhan Li, Le Zhang, Fangming Liu, Guilan Fan, Fangyi Cheng*

Key Laboratory of Advanced Energy Materials Chemistry (Ministry of Education), Renewable Energy Conversion and Storage Center (RECAST), College of Chemistry, Nankai University, Tianjin 300071, China

Correspondence should be addressed to Zhenhua Yan; [yzh@nankai.edu.cn](mailto:yzh@nankai.edu.cn) and Fangyi Cheng; [fycheng@nankai.edu.cn](mailto:fycheng@nankai.edu.cn)


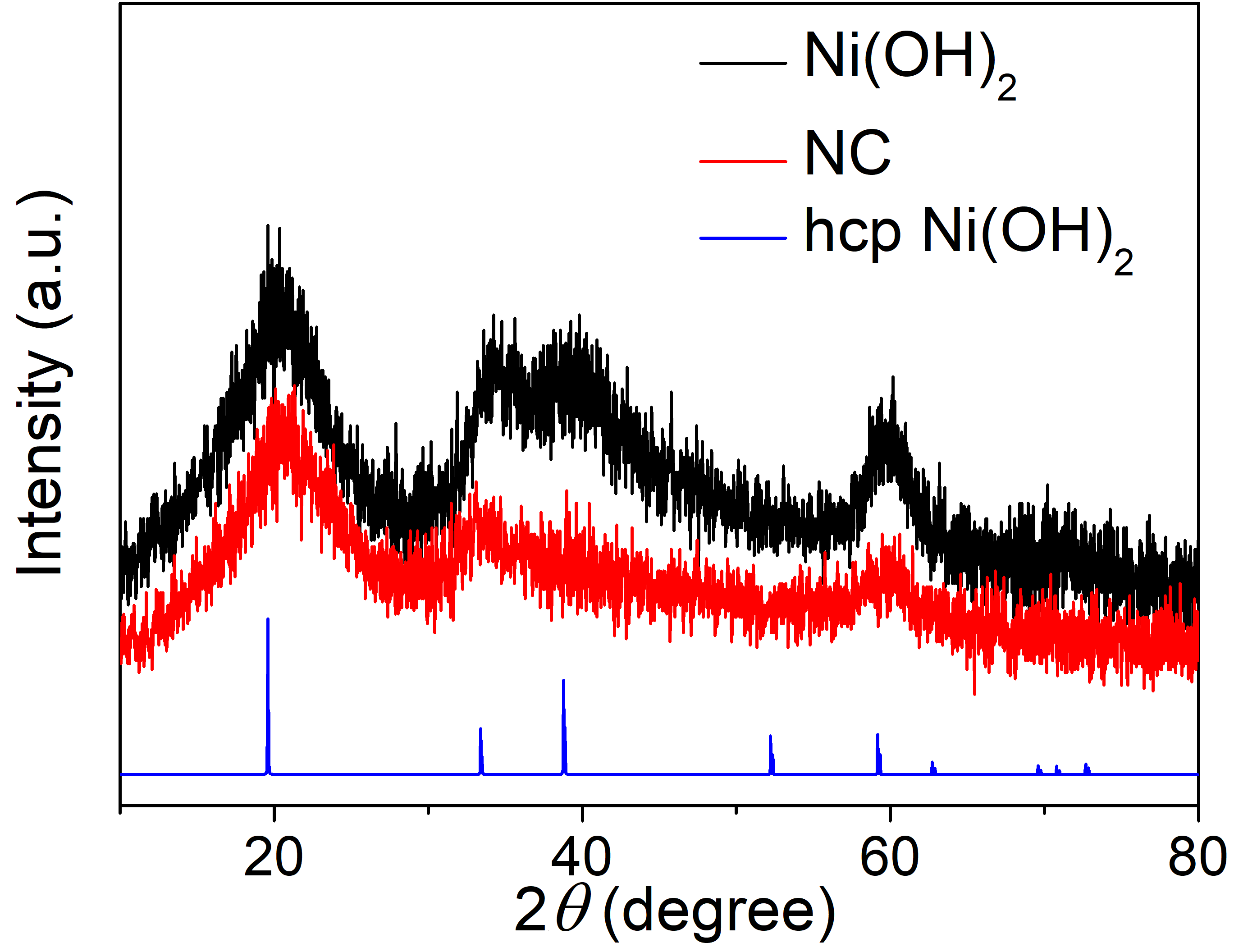


**Figure S1.** Powder XRD patterns of Ni(OH)_2_ and NC scratched down from the Ti mesh.


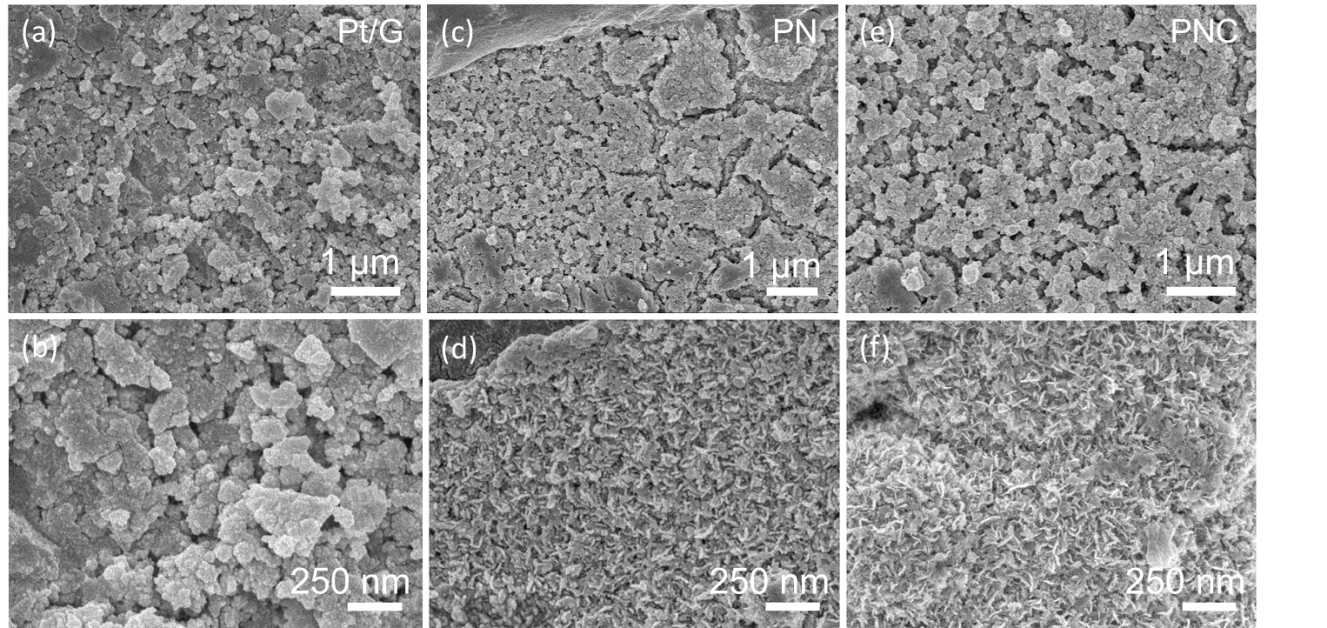


**Figure S2.** SEM images of Pt/G (a, b), PN (c, d), and PNC (e, f) at low (a,c,e) and high (b,d,f) magnification.


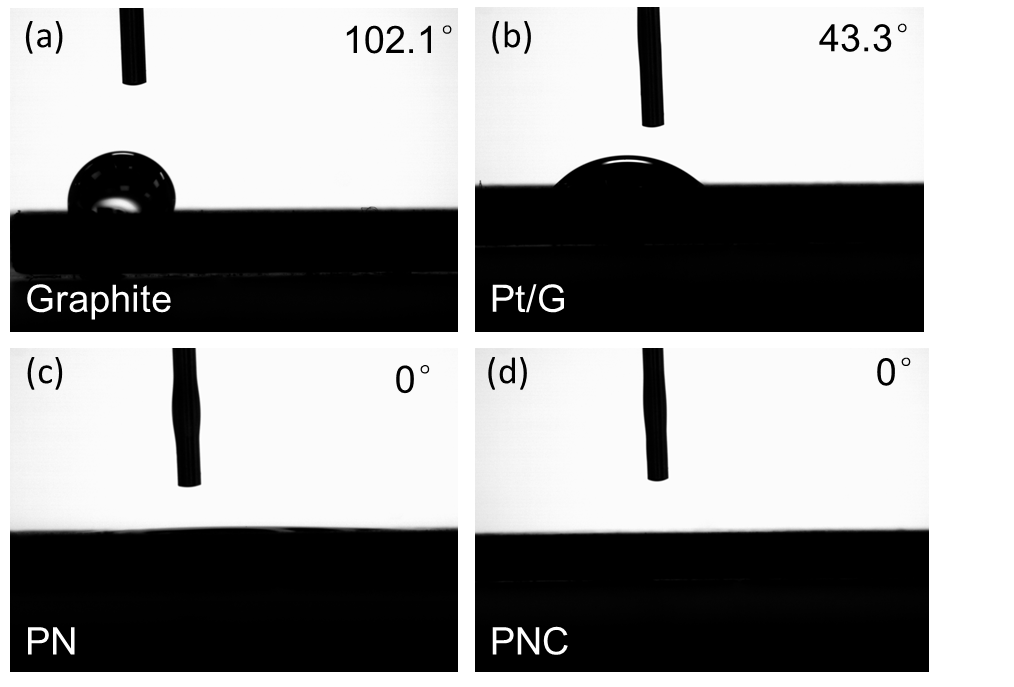


**Figure S3.** Contact angles measured on graphite substrate (a) before and (b-d) after the electrodeposition of Pt (b), PN (c), and PNC (d).


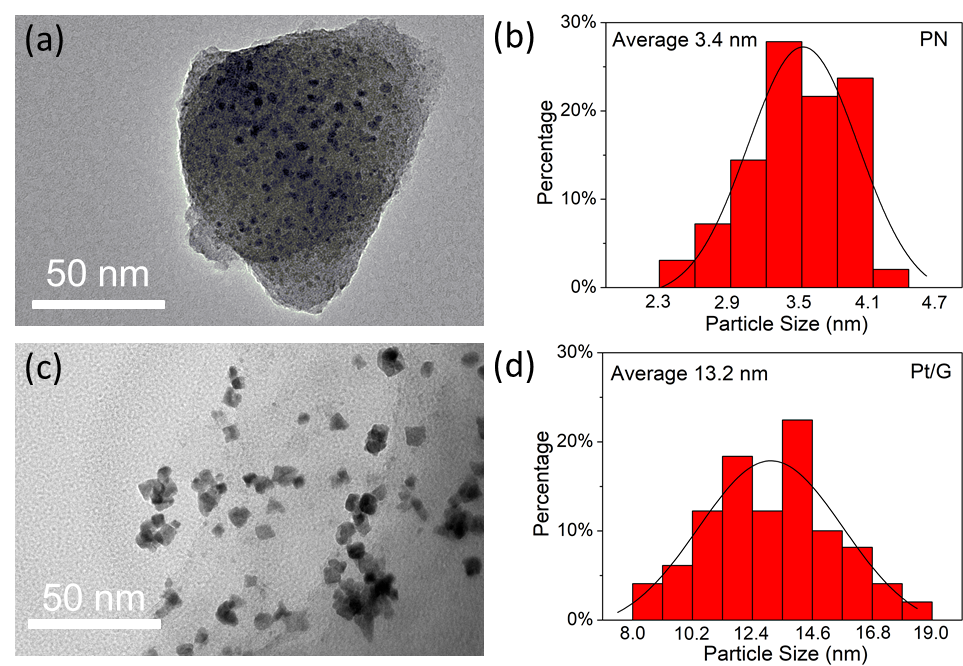


**Figure S4.** (a,b) TEM images and particle size distribution of PN. (c,d) TEM images and particle size distribution of Pt/G.


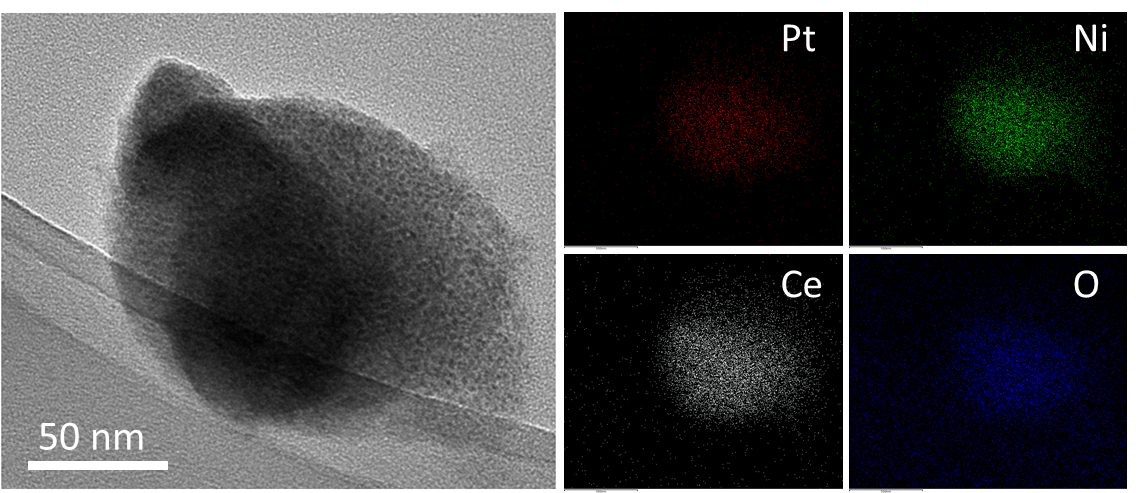


**Figure S5.** Elemental mapping images of PNC.


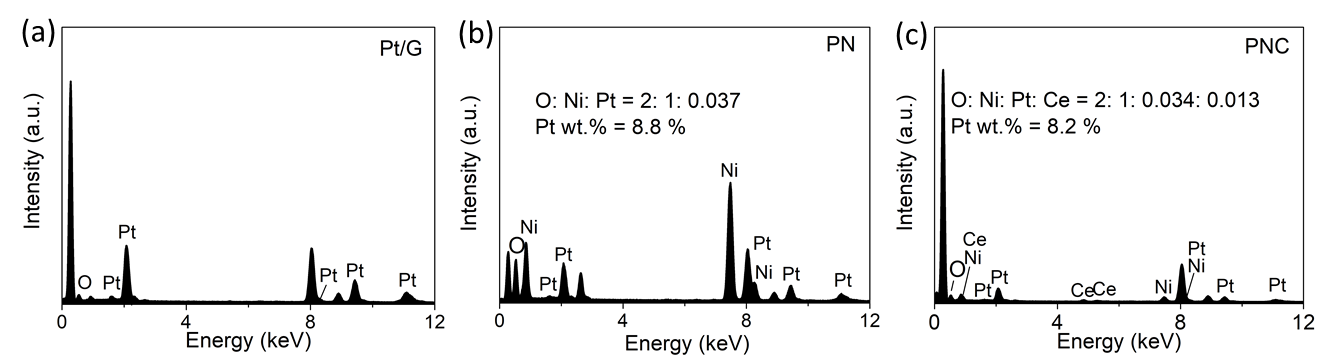


**Figure S6.** EDS spectra of (a) Pt, (b) PN and (c) PNC.


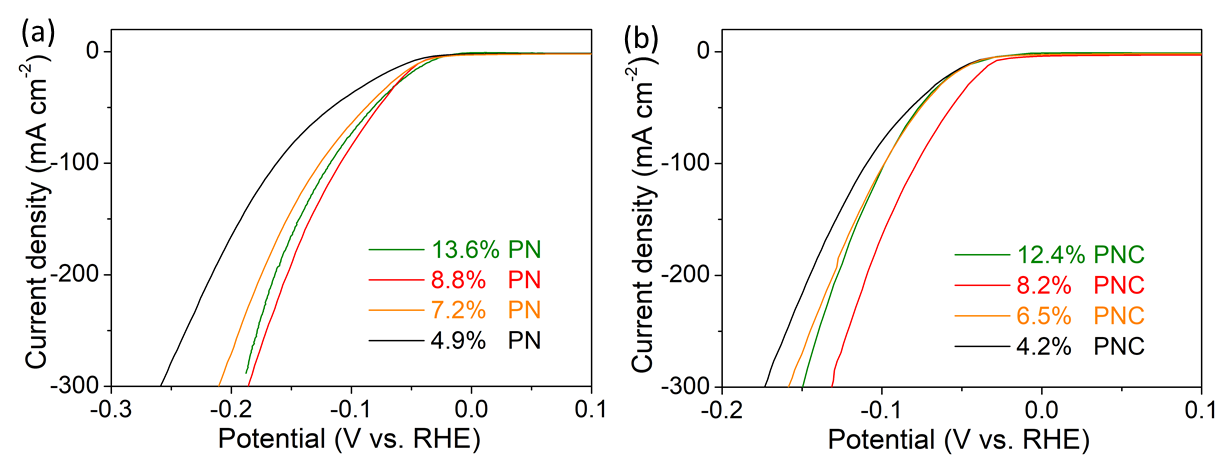


**Figure S7.** HER polarization curves of (a) PN and (b) PNC hybrid with different Pt loadings.


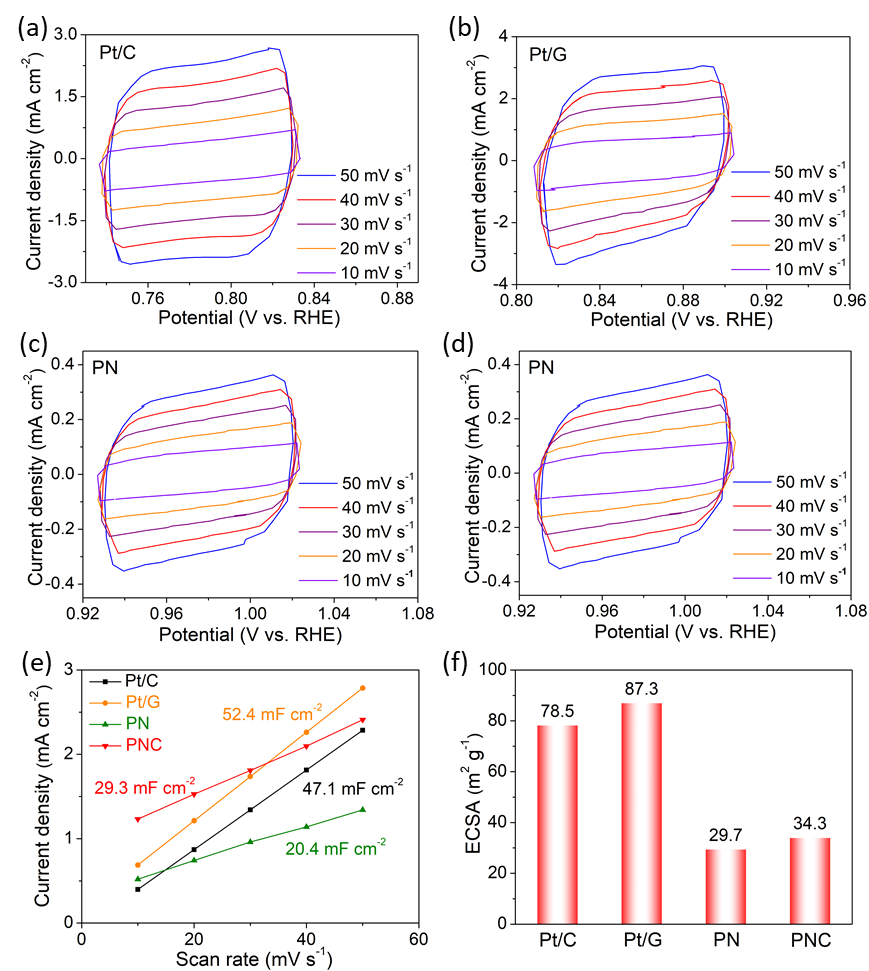


**Figure S8.** CV curves of (a) Pt/C, (b) electrodeposited Pt, (c) PN, and (d) PNC. (e) The corresponding linear fitting of the current density versus scan rates. (f) Comparison of ECSA.


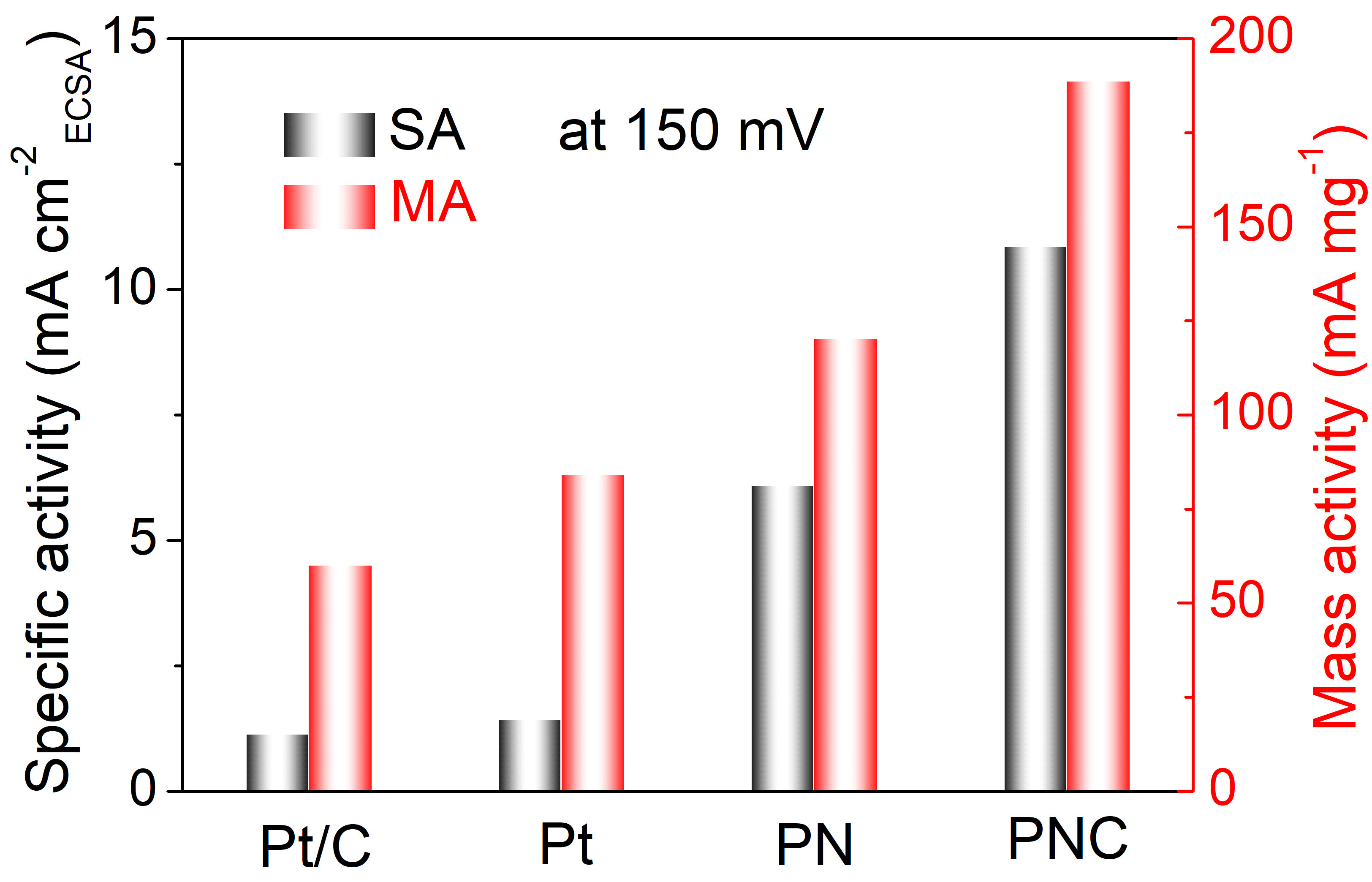


**Figure S9.** Specific and mass activities derived from the HER polarization curves at 150 mV.


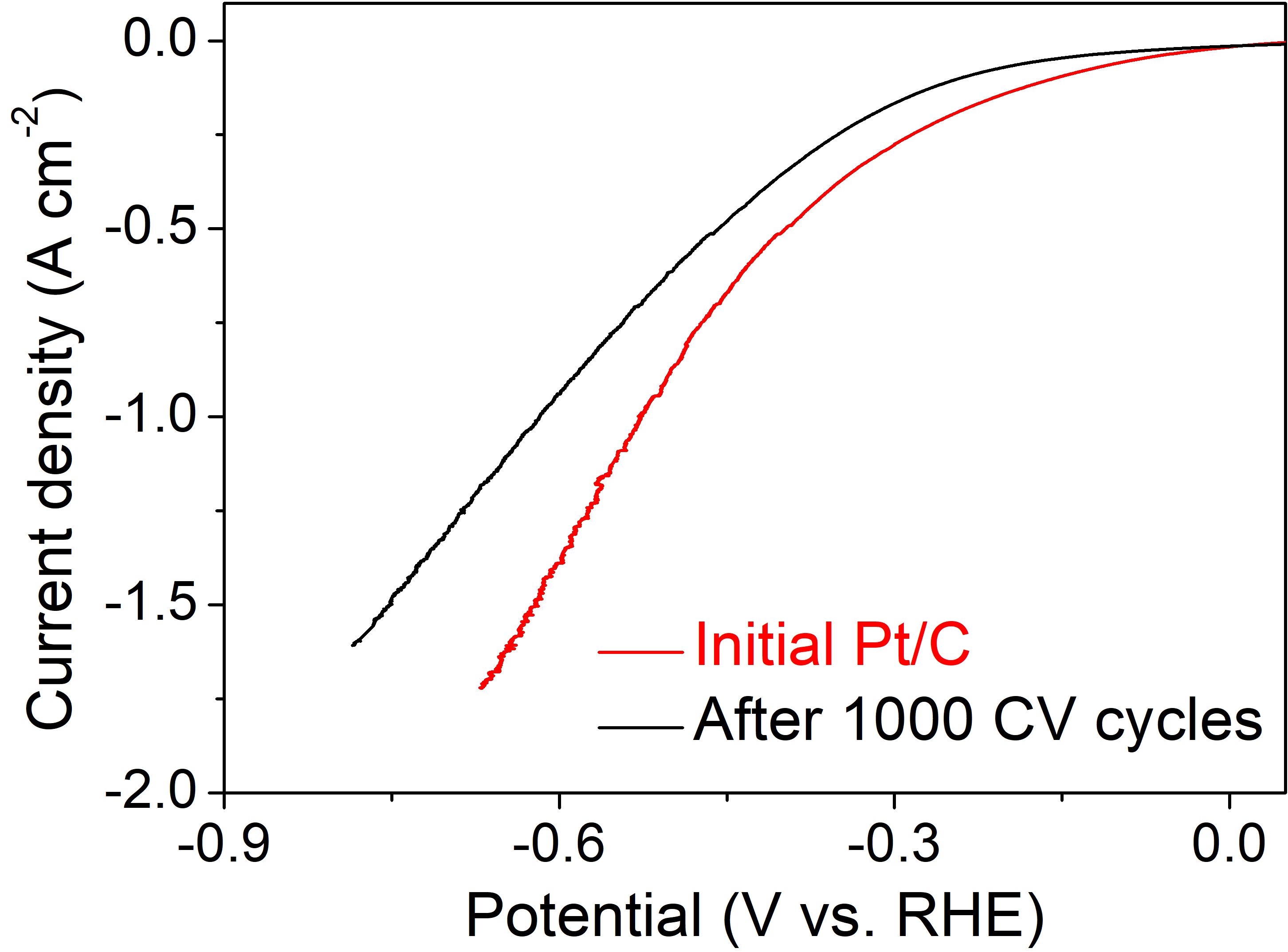


**Figure S10.** Polarization curves of 20 wt.% Pt/C before and after 1000 HER cycles.


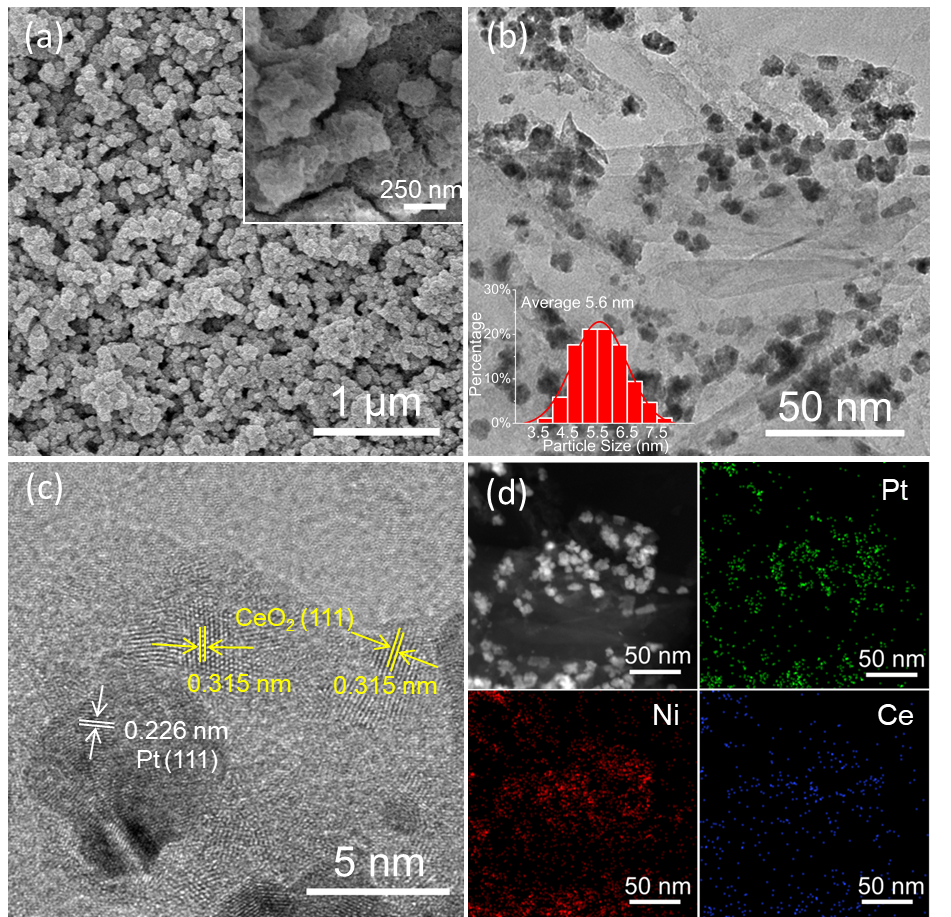


**Figure S11.** (a) SEM, (b) TEM, (c) HRTEM and (d) elemental mapping images of PNC after 70 h of HER stability test at 1000 mA cm^−2^. Insets (a) and (b) shows high-magnification SEM image, and size distribution of Pt nanoparticles, respectively.


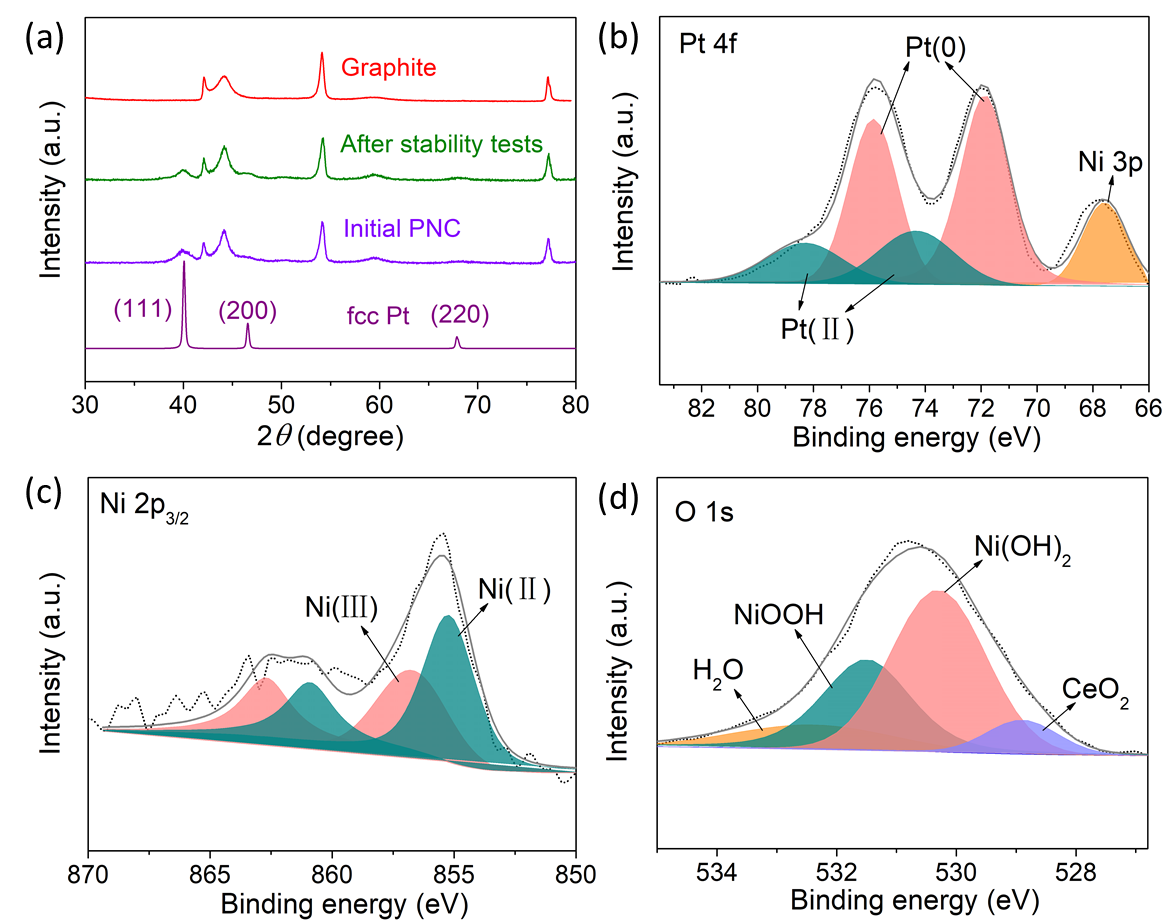


**Figure S12.** (a) XRD pattern of PNC after HER stability test for 70 h at 1000 mA cm^−2^. High-resolution XPS spectra of (b) Pt 4f, (c) Ni 2p3/2 and (d) O 1s of PNC after stability test.


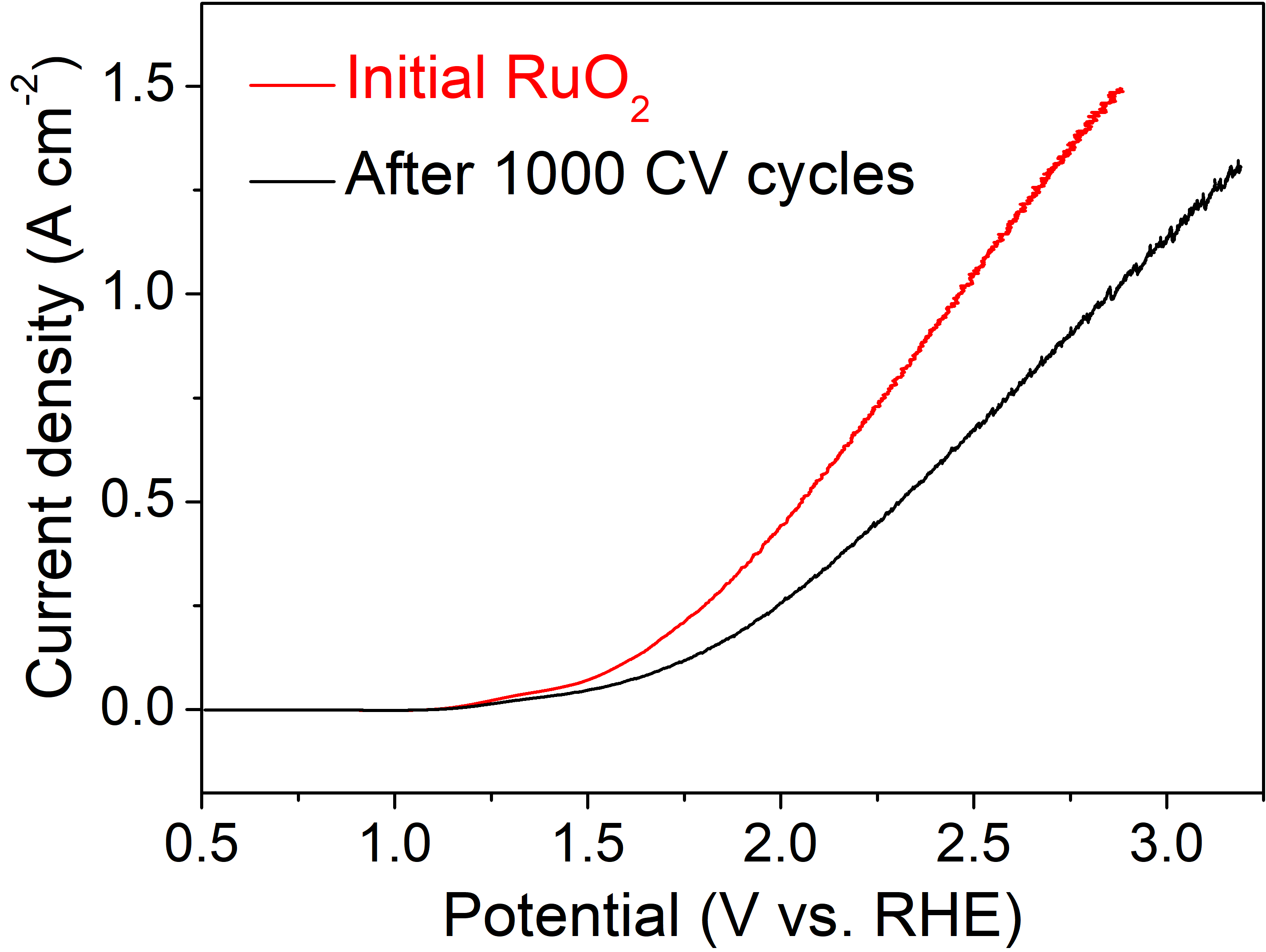


**Figure S13.** Polarization curves of RuO_2_ before and after 1000 OER cycles.


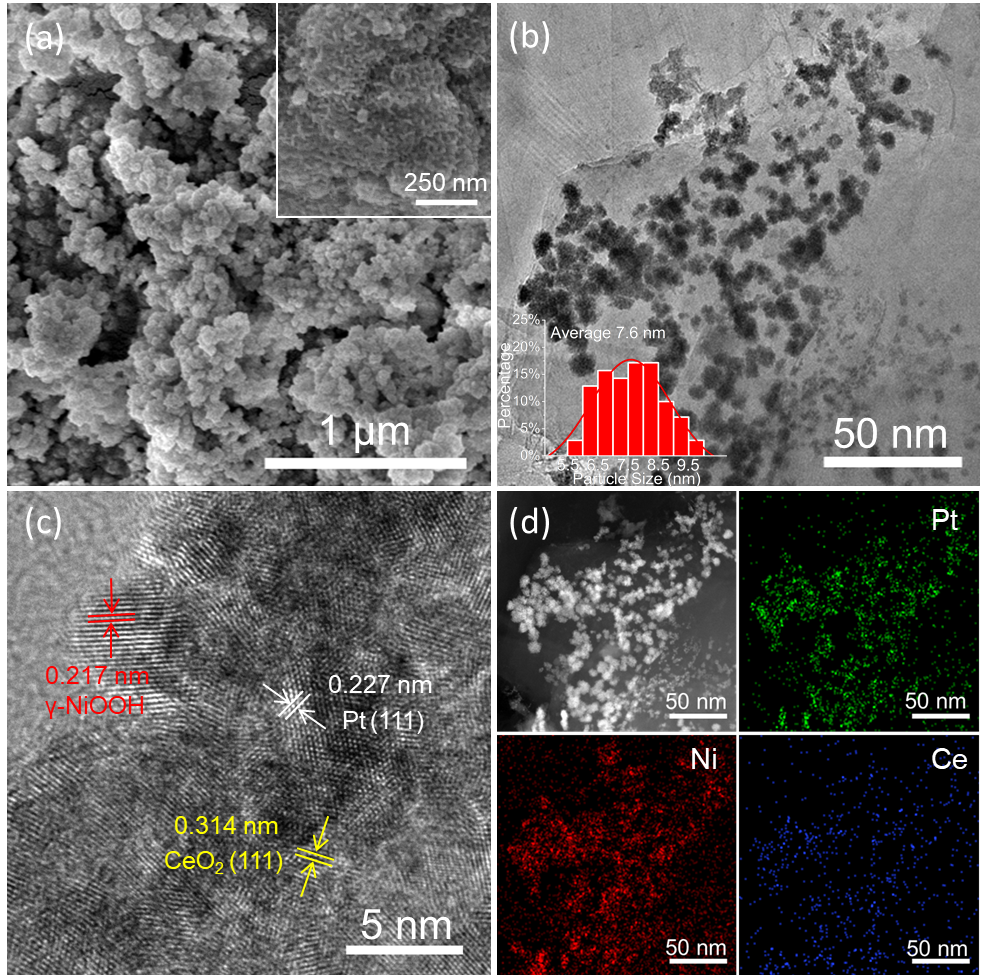


**Figure S14.** (a) SEM, (b) TEM, (c) HRTEM and (d) elemental mapping images of PNC after 70 h of OER stability test at 1000 mA cm^−2^. Insets (a) and (b) shows high-magnification SEM image and size distribution of Pt nanoparticles, respectively.


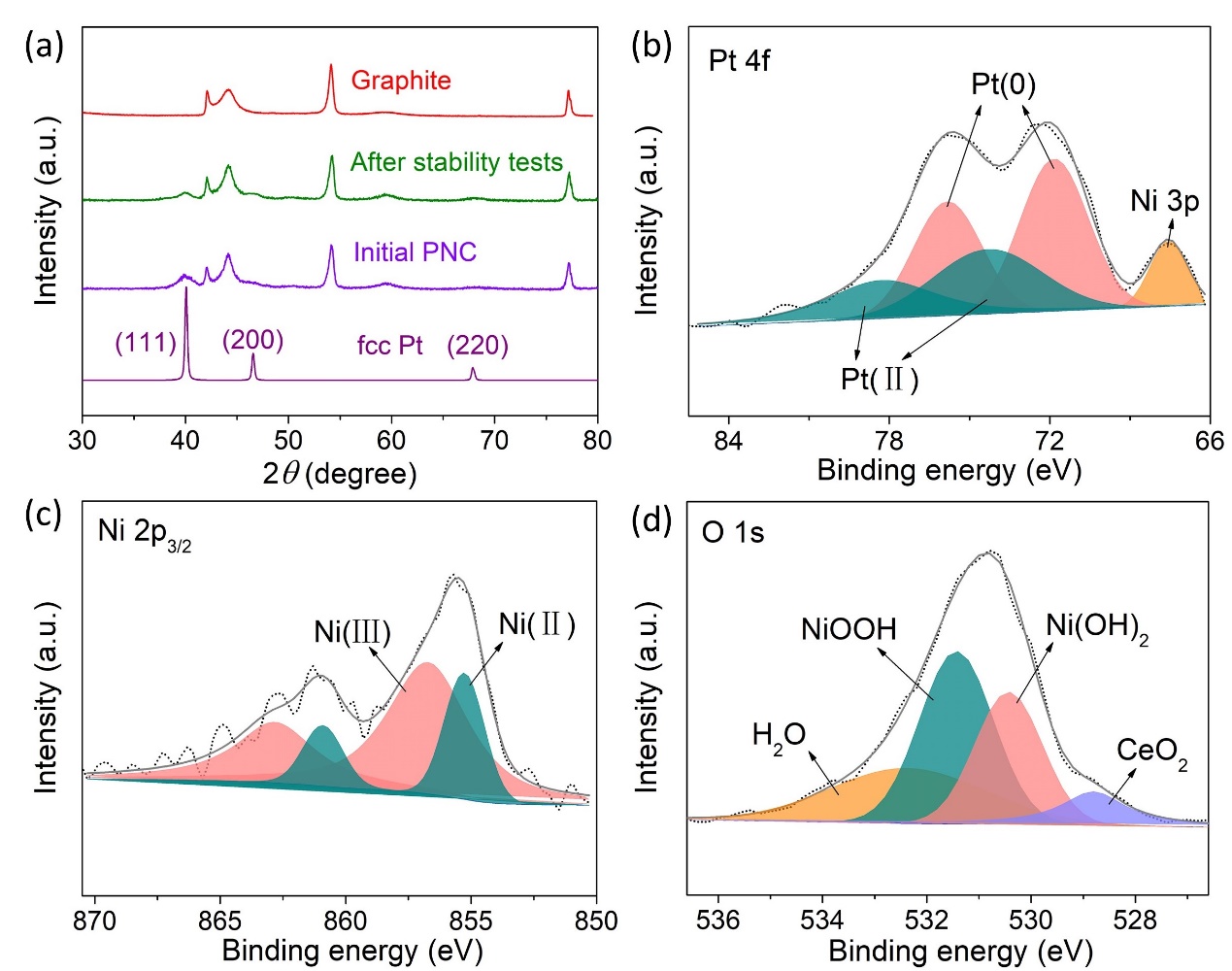


**Figure S15.** (a) XRD patterns of PNC after OER stability test. High-resolution XPS spectra of (b) Pt 4f, (c) Ni 2p3/2 and (d) O 1s regions of PNC after stability test.


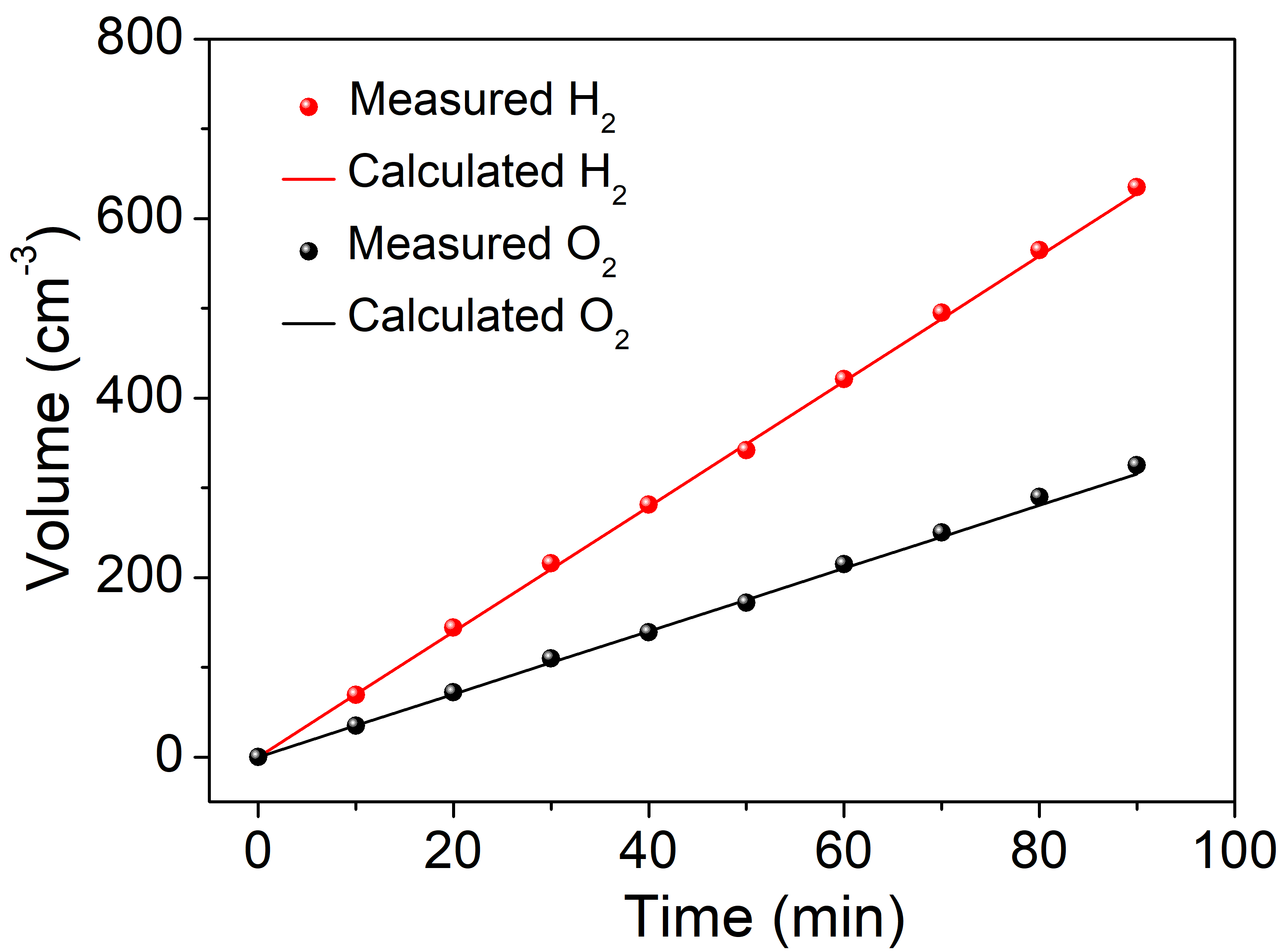


**Figure S16.** Generated and theoretical volumes of H_2_ and O_2_ over time at a constant current density of 1000 mA cm^−2^ in PNC || PNC electrolyzer.


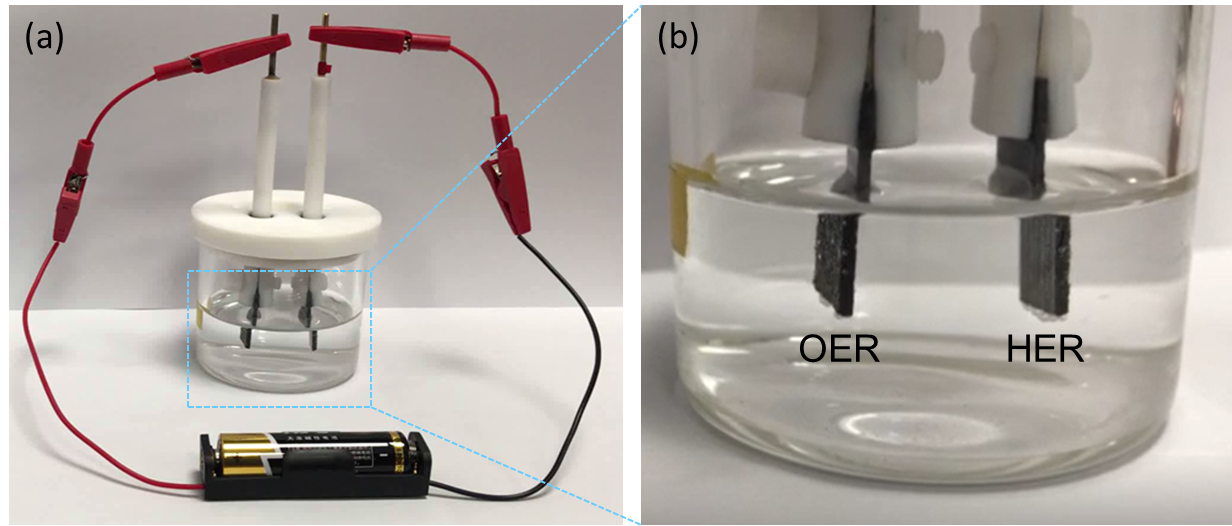


**Figure S17.** Digital photographs of the PNC || PNC electrolyzer powered by a 1.5 V battery.

**Table S1.** The Pt contents in PN and PNC with different electrodeposition time in H_2_PtCl_6_.

| Electrodeposition time (s) | 50 | 100 | 150 | 200 |
| --- | --- | --- | --- | --- |
| Pt content (wt.%) in PN | 4.9 | 7.2 | 8.8 | 13.6 |
| Pt content (wt.%) in PNC | 4.2 | 6.5 | 8.2 | 12.4 |

**Table S2.** Comparison of HER catalytic performances of PNC with other electrocatalysts.

| Materials | Pt loading  (mg cm^–2^) | Electrolyte | Overpotential  (mV vs. RHE)  @4 mA cm^–2^ | Current density  (mA cm^–2^)  @−70 mV vs. RHE | Mass activity  (mA mg^–1^_Pt_)  @−70 mV vs. RHE | Durability (h) under current density  (mA cm^–2^) | Reference |
| --- | --- | --- | --- | --- | --- | --- | --- |
| PNC | 0.041 | 1 M KOH | 23.0 | 66 | 1707 | 70 (1000) | This work |
| Ni(OH)_2_/Pt–islands/  Pt(111) surface | - | 0.1 M KOH+  10^–3^ M LiOH | ~110 | ~2.2 | - | - | Ref. 1 |
| Pt NWs/SL–Ni(OH)_2_ | 0.016 | 1 M KOH | 85.0 | 11 | 679 | 11 (~4) | Ref. 2 |
| PtNi nanoframes/Ni(OH)_2_ | 0.0051 | 0.1 M KOH | - | 6.5 | 300 | - | Ref. 3 |
| NiO_x_/Pt_3_Ni  Pt_3_Ni_3_-NWs | 0.015 | 1 M KOH | - | 40 | 2600 | 3 (~40) | Ref. 4 |
| Pt_3_Ni_2_-NWs–S/C | 0.015 | 1 M KOH | - | 37 | 2430 | 5 (5) | Ref. 5 |
| Pt/C/10 wt.%  SL Ni(OH)_2_ | 0.0011 | 0.1 M KOH | 106 | 2.3 | 2017 | - | Ref. 6 |
| Ni_3_N/Pt | 0.30 | 1 M KOH | 37.0 | 40 | 130 | 24 (50) | Ref. 7 |
| Pt/Co(OH)_2_–CC | 0.39 | 1 M KOH | - | 30 | 76 | 20 (10) | Ref. 8 |
| Ni(OH)_2_–PtO_2_ NS/Ti | - | 1 M KOH | 31.4 | - | - | 100 (20) | Ref. 9 |
| Pt@2D-Ni(OH)_2_ | 0.0011 | 0.1 M KOH | 87.0 | - | - | 5.5 (~4) | Ref. 10 |
| Pt/Ni(HCO_3_)_2_ | 0.008 | 1 M KOH | ~43 | ~70 | - | 3.3 (10) | Ref. 11 |
| Pt/Ni(OH)_2_/NF-A | 0.229 | 1 M KOH | ~18 | ~36 | 157 | 20 (10) | Ref. 12 |
| Pt-Ni(OH)_2_-2h-NF20 | - | 1 M KOH | ~50 | ~5 | - | 50 (10) | Ref. 13 |

**Table S3.** Comparison of OER catalytic performances of PNC with other electrocatalysts.

| Materials | Loading mass  (mg cm^–2^) | Electrolyte | Overpotential  (mV vs. RHE)  @10 mA cm^–2^ | Tafel slope  (mV dec^–1^) | Durability (h) under current density  (mA cm^–2^) | Reference |
| --- | --- | --- | --- | --- | --- | --- |
| PNC | 0.50 | 1 M KOH | 209 | 54 | 70 (1000) | This work |
| NiFe/NF | - | 1 M KOH | 215 | 28 | 10 (100) | Ref. 14 |
| NiFe LDH/GO | 0.25 | 1 M KOH | 210 | 39 | 8 (10) | Ref. 15 |
| NiCo–MOF/Cu Foam | 0.20 | 1 M KOH | 189 | 42 | 200 (10) | Ref. 16 |
| NiCeO_x_–Au | - | 1 M KOH | 290 | - | 2 (10) | Ref. 17 |
| ECPT–Co@C | 0.040 | 1 M KOH | 333 | 58 | 8 (20) | Ref. 18 |
| RuO_2_ | 0.040 | 1 M KOH | 350 | 94 | 8 (20) | Ref. 18 |
| NiFe-NS | 0.070 | 1 M KOH | 300 | 40 | 13 (10) | Ref. 19 |
| IrO_x_ | 0.21 | 1 M KOH | 340 | 45 | 6 (10) | Ref. 19 |
| NiFe-LDH | 0.25 | 1 M KOH | 247 | 31 | 108 (20) | Ref. 20 |
| Ir/C-20 wt.% | 0.25 | 1 M KOH | 270 | 40 | 2 (20) | Ref. 20 |
| FeCoW/Au Foam | 0.21 | 1 M KOH | 191 | 37 | 550 (30) | Ref. 21 |
| Ni_4_Ce_1_@CP | - | 1 M KOH | 220 | 82 | 20 (20) | Ref. 22 |
| Ce-NiO-E | - | 1 M KOH | 382 | 118 | - | Ref. 23 |
| NiCe@NiFe | - | 1 M KOH | 220 | 60 | - | Ref. 24 |

**Table S4.** Comparison of the performance of PNC and other bifunctional electrocatalyst in symmetric electrolyzers with 1.0 M KOH electrolyte.

| Catalyst | Current density  (j, mA cm^–2^) | Overall voltage  (V) | Durability (h)  under current  density (mA cm^–2^) | Reference |
| --- | --- | --- | --- | --- |
| PNC | 10 | 1.45 | 85 (1000) | This work |
| Ni_2_P/Ni/Ni foam | 10 | 1.49 | 100 (10) | Ref. 25 |
| Cu@CoS_x_/CF | 10 | 1.50 | 200 (100) | Ref. 26 |
| Ni–ZIF/Ni–B@NF | 10 | 1.54 | 64 (10) | Ref. 27 |
| Pt/CoS_2_/CC | 10 | 1.55 | 20 (10) | Ref. 28 |
| MoS_2_/Ni_3_S_2_ | 10 | 1.56 | 10 (10) | Ref. 29 |
| Mo–NiCo_2_O_4_/Co_5.47_N/NF | 10 | 1.56 | 12 (10) | Ref. 30 |
| Ni@NC-800 | 10 | 1.60 | 50 (~17) | Ref. 31 |
| O–CoP | 10 | 1.60 | 18 (15) | Ref. 32 |
| FeNi_3_N/NF | 10 | 1.62 | 450 (10) | Ref. 33 |
| Co_9_S_8_ nanowires@NiCo LDH | 10 | 1.63 | 12 (10) | Ref. 34 |
| NiSe/NF | 10 | 1.63 | 20 (20) | Ref. 35 |
| Ni_5_P_4_–NiP_2_ nanosheets/NF | 10 | 1.64 | 50 (10) | Ref. 36 |
| NiCo_2_O_4_ hollow microcuboids | 10 | 1.65 | 36 (20) | Ref. 37 |
| Ni–P film | 10 | 1.67 | 24 (10) | Ref. 38 |

**Supplementary References**

[1] R. Subbaraman, D. Tripkovic, N. M. Markovic et al. “Enhancing hydrogen evolution activity in water splitting by tailoring Li^+^−Ni(OH)_2_−Pt interfaces,” *Science*, vol. 334, no. 6060, pp. 1256-1260, 2011.

[2] H. J. Yin, S. L. Zhao, Z. Y. Tang et al. “Ultrathin platinum nanowires grown on single-layered nickel hydroxide with high hydrogen evolution activity,” *Nature Communications*, vol. 6, article 6430, 2015.

[3] C. Chen, Y. Kang, V. R. Stamenkovic et al. “Highly crystalline multimetallic nanoframes with three-dimensional electrocatalytic surfaces,” *Science*, vol. 343, no. 6177, pp. 1339-1343, 2014.

[4] P. T. Wang, J. L. Yao, X. Q. Huang et al. “Phase and interface engineering of platinum–nickel nanowires for efficient electrochemical hydrogen evolution,” *Angewandte Chemie International Edition*, vol. 55, no. 41, pp. 12859-12863, 2016.

[5] P. T. Wang, S. J. Guo, X. Q. Huang et al. “Precise tuning in platinum–nickel/nickel sulfide interface nanowires for synergistic hydrogen evolution catalysis,” *Nature Communications*, vol. 8, article 14580, 2017.

[6] L. Wang, L. Shi, J. Jin et al. “Optimizing the volmer step by single-layer nickel hydroxide nanosheets in hydrogen evolution reaction of platinum,” *ACS Catalysis*, vol. 5, no. 6, pp. 3801-3806, 2015.

[7] Y. H. Wang, Y. G. Wang, G. F. Zheng et al. “Superb alkaline hydrogen evolution and simultaneous electricity generation by Pt‐decorated Ni_3_N nanosheets,” *Advanced Energy Materials*, vol. 7, no. 2, article 1601390, 2017.

[8] Z. X. Xing, C. Han, X. R. Yang et al. “Ultrafine Pt nanoparticle-decorated Co(OH)_2_ nanosheet arrays with enhanced catalytic activity toward hydrogen evolution,” *ACS Catalysis*, vol. 7, no. 10, pp. 7131-7135, 2017.

[9] L. S. Xie, X. P. Sun, L. Chen et al. “A Ni(OH)_2_–PtO_2_ hybrid nanosheet array with ultralow Pt loading toward efficient and durable alkaline hydrogen evolution,” *Journal of Material Chemistry A*, vol. 6, no. 5, pp. 1967-1970, 2018.

[10] L. Wang, C. Wang, J. Jin et al. “Platinum–nickel hydroxide nanocomposites for electrocatalytic reduction of water,” *Nano Energy*, vol. 31, pp. 456-461, 2017.

[11] M. M. Lao, K. Rui, W. P. Sun et al. “Platinum/nickel bicarbonate heterostructures towards

accelerated alkaline hydrogen evolution reaction.” *Angewandte Chemie International Edition,* vol. 58, no. 16, pp. 5432-5437, 2019.

[12] Y. F. Gu, Y. Q. Wang, Y. Men et al. “Well-dispersed Pt nanodots interfaced with Ni(OH)_2_ on anodized nickel foam for efficient hydrogen evolution reaction.” *International Journal of Hydrogen Energy*, DOI: 10.1016/j.ijhydene.2020.07.047, 2020.

[13] Q. F. Liu, Z. Yan, G. Q. Sun et al. “Optimizing platinum location on nickel hydroxide nanosheets to accelerate the hydrogen evolution reaction.” *ACS Applied Materials & Interfaces*, vol. 12, pp. 24683-24692, 2020.

[14] X. Y. Lu, C. Zhao. “Electrodeposition of hierarchically structured three-dimensional nickel–iron electrodes for efficient oxygen evolution at high current densities,” *Nature Communications*, vol. 6, article 6616, 2015.

[15] X. Long, J. K. Li, S. H. Yang et al. “A strongly coupled graphene and FeNi double hydroxide hybrid as an excellent electrocatalyst for the oxygen evolution reaction,” *Angewandte Chemie International Edition*, vol. 53, no. 29, pp. 7584-7588, 2014.

[16] S. L. Zhao, H. J. Zhao, Z. Y. Tang et al. “Ultrathin metal–organic framework nanosheets for electrocatalytic oxygen evolution,” *Nature Energy*, vol. 1, article 16184, pp. 1-10, 2016.

[17] D. Ng, A. Vojvodic, T. F. Jaramillo et al. “Gold-supported cerium-doped NiO_x_ catalysts for water oxidation,” *Nature Energy*, vol. 1, article 16053, 2016.

[18] Q. Q. Xiao, Y. X. Zhang, Y. M. Yang et al. “A high-performance electrocatalyst for oxygen evolution reactions based on electrochemical post-treatment of ultrathin carbon layer coated cobalt nanoparticles,” *Chemical Communications*, vol. 50, no. 86, pp. 13019-13022, 2014.

[19] F. Song, X. L. Hu. “Exfoliation of layered double hydroxides for enhanced oxygen evolution catalysis,” *Nature Communications*, vol. 5, article 4477, 2014.

[20] M. Gong, Y. G. Li, H. J. Dai et al. “An advanced Ni–Fe layered double hydroxide electrocatalyst for water oxidation,” *Journal of the American Chemical Society*, vol. 135, no. 23, pp. 8452-8455, 2013.

[21] B. Zhang, X. L. Zheng, O. Voznyy et al. “Homogeneously dispersed multimetal oxygen-evolving catalysts,” *Science*, vol. 352, no. 6283, pp. 333-337, 2016.

[22] D. D. Zhao, Y. C. Pi, X. Q. Huang et al. “Enhancing oxygen evolution electrocatalysis via the intimate hydroxide-oxide interface.” ACS nano, vol. 12, pp. 6245-6251, 2018.

[23] W. Gao, Z. M. Xia, Y. Q. Qu et al. “Comprehensive understanding of the spatial configurations of CeO2 in NiO for the electrocatalytic oxygen evolution reaction: embedded or surface-loaded.” Advanced Functional Materials, vol. 28, article 1706056, 2018.

[24] G. Liu, M. H. Wang, J. P. Li et al. “3D porous network heterostructure NiCe@NiFe electrocatalyst for efficient oxygen evolution reaction at large current densities.” Applied Catalysis B: Environmental, vol. 260, article 118199, 2020.

[25] B. You, N. Jiang, Y. J. Sun et al. “Hierarchically porous urchin-like Ni_2_P superstructures supported on nickel foam as efficient bifunctional electrocatalysts for overall water splitting,” *ACS Catalysis*, vol. 6, no. 2, pp. 714-721, 2016.

[26] Y. P. Liu, Y. Zhang, X. X. Zou et al. “Coupling sub-nanometric copper clusters with quasi-amorphous cobalt sulfide yields efficient and robust electrocatalysts for water splitting reaction,” *Advanced Materials*, vol. 29, no. 13, article 1606200, 2016.

[27] H. B. Xu, B. Fei, R. B. Wu et al. “Boronization‐induced ultrathin 2D nanosheets with abundant crystalline–amorphous phase boundary supported on nickel foam toward efficient water splitting.” *Advanced Energy Materials*, vol. 10, article 1902714, 2020.

[28] X. P. Han, C. Zhong, W. B. Hu et al. “Ultrafine Pt nanoparticle-decorated pyrite-type CoS_2_ nanosheet arrays coated on carbon cloth as a bifunctional electrode for overall water splitting,” *Advanced Energy Materials*, vol. 8, article 1800935, 2018.

[29] J Zhang, T. Wang X. L. Feng et al. “Interface engineering of MoS_2_/Ni_3_S_2_ heterostructures for highly enhanced electrochemical overall-water-splitting activity,” *Angewandte Chemie International Edition*, vol. 55, no. 23, pp. 6702-6707, 2016.

[30] W. X. Liu, L. H. Yu, X. H. Cao et al. “Non–3d metal modulation of a 2D Ni–Co heterostructure array as multifunctional electrocatalyst for portable overall water splitting.” Small, vol. 16, no. 10, article 1906775, 2020.

[31] Y. Xu, W. G. Tu, R. Xu et al. “Nickel nanoparticles encapsulated in few-layer nitrogen-doped graphene derived from metal-organic frameworks as efficient bifunctional electrocatalysts for overall water splitting,” *Advanced Materials*, vol. 29, no. 11, p. 1605957, 2017.

[32] G. Y. Zhou, M. Li, Y. W. Tang et al. “Regulating the electronic structure of CoP nanosheets by O incorporation for high–efficiency electrochemical overall water splitting.” *Advanced Functional Materials*, vol. 30, article 1905252, 2020.

[33] B. Zhang, C. H. Xiao, S. M. Xie et al. “Iron–nickel nitride nanostructures in situ grown on surface-redox-etching nickel foam: efficient and ultrasustainable electrocatalysts for overall water splitting,” *Chemistry of Materials*, vol. 28, no. 19, pp. 6934-6941, 2016.

[34] J. G. Yan, L. G. Chen, X. Liang. “Co_9_S_8_ nanowires@NiCo LDH nanosheets arrays on nickel foams towards efficient overall water splitting,” *Science Bulletin*, vol. 64, no. 3, pp. 158-165, 2019.

[35] C. Tang C, N. Y. Cheng, X. P. Sun et al. “NiSe nanowire film supported on nickel foam: an efficient and stable 3D bifunctional electrode for full water splitting,” *Angewandte Chemie International Edition*, vol. 54, no. 32, pp. 9351-9355, 2015.

[36] X. G. Wang, W. Li, L. F. Liu et al. “Fast fabrication of self-supported porous nickel phosphide foam for efficient, durable oxygen evolution and overall water splitting,” *Journal of Materials Chemistry A*, vol. 4, no. 15, pp. 5639-5646, 2016.

[37] X. H. Gao, X. W. Zhang, Z. Lin et al. “Hierarchical NiCo_2_O_4_ hollow microcuboids as bifunctional electrocatalysts for overall water-splitting,” *Angewandte Chemie International Edition*, vol. 55, no. 21, pp. 6290-6294, 2016.

[38] N. Jiang, B. You, Y. J. Sun et al. “Bifunctionality and mechanism of electrodeposited nickel-phosphorous films for efficient overall water splitting,” *ChemCatChem*, vol. 8, no. 1, pp. 106-112, 2016.
